# Naphthalimide-based conjugated macrocycles possessing tunable self-assembly and supramolecular binding behaviours

**DOI:** 10.3389/fchem.2022.1094828

**Published:** 2022-12-20

**Authors:** Dongyue An, Yutao Sun, Dongdong Chang, Jiangyu Zhu, Shumin Chen, Xuefeng Lu

**Affiliations:** ^1^ Department of Materials Science, State Key Laboratory of Molecular Engineering of Polymers, Fudan University, Shanghai, China; ^2^ School of Mathematics and Physics, Jingchu University of Technology, Jingmen, China

**Keywords:** macrocycle, electron acceptors, 1,8-naphthalimide, self-assembly, supramolecular binding

## Abstract

The special geometric configurations and optoelectronic properties of *p*-conjugated macrocycles have always been the focus of materials science. The incorporation of building moieties with different features into macrocycles can not only change their geometric configurations, but also realize the regulation of intramolecular charge transfer, which is expected to bring unusual performance in supramolecular chemistry and optoelectronic devices. Herein, four novel *p*-conjugated macrocycles based on typical electron acceptor units naphthalimide (NMI) with aryl or alkyl substitutions were reported. The different substitutions on NMI had greatly affected the self-assembly behaviours of these macrocycles. Alkyl substituted **NP2b** and **NP3b** showed obvious self-aggregation in solution, while similiar phenomenon was not found in aryl substituted macrocycles **NP2a** and **NP3a**, which can be attributed to the steric hindrance caused by rigid aryl groups that could affect the aggregation of macrocycles in solution. In addition, all the macrocycles exhibited supramolecular encapsulation with C_70_, in which the larger macrocycles **NP3a** and **NP3b** with twisted geometries showed stronger binding affinity towards C_70_ than the corresponding small-size macrocycles **NP2a** and **NP2b** with near-planar geometries. Our studies have greatly extended the family of macrocycles based on NMI, pointing out the direction for further supramolecular studies and applications on *p*-conjugated macrocycles.

## Introduction

Supramolecular chemistry is a class of science that focuses on non-covalent interactions between molecules, such as hydrogen bonds, electrostatic interactions, hydrophobic interactions and *p*-π interactions (Ringsdorf and Simon, 1994; Whitesides and Grzybowski, 2002). These weak interactions give rise to host-guest binding and self-assembly behaviour. Among them, the host-guest interaction refers to the process in which two or more kinds of molecules, namely the host and the guest molecule, are combined by non-covalent bonds (Yang et al., 2015; Li et al., 2020), while self-assembly is the process in which molecules or parts of molecules form ordered aggregates spontaneously (Zhou and Yan, 2009; Song et al., 2021). These supramolecular complexes formed by non-covalent interactions can realize molecular recognition, catalysis, reaction, transfer and other functions (Park and Simmons, 1968; Kim et al., 2019; Dey et al., 2021) (Ghosh et al., 2018) (Ghosh et al., 2022) (Kumar et al., 2022), which have important theoretical significance and broad application prospects in materials science, information science and life science. We pay special attention to the supramolecular properties of *p*-conjugate system. However, the *p*-conjugate molecules with supramolecular properties reported so far are mostly simple small molecules, such as carbazol (Yang et al., 2008), hexabenzocoronene (HBC) (Dou et al., 2008), porphyrin (Pp) (Wolffs et al., 2005; Hou et al., 2006; Helmich et al., 2010) and perylene bisimide (PBI) (Würthner et al., 2008). Therefore, the key to supramolecular research is to develop novel *p*-conjugated materials with unique geometric structure, good solubility, versatile functionality, homologues and host–guest ability (Ogoshi et al., 2016).

The *p*-conjugated macrocycles (Savage, 2011; Loh et al., 2016) are a very special class of conjugated small molecules, which can be regarded as cyclic conjugated oligomers with definite diameters (Kudernac et al., 2009). Thanks to their special annular geometries, especially the existence of inner and outer rings, conjugated macrocycles exhibit unique physicochemical properties that distinguish them from other small molecules and linearly conjugated system cavities, such as photophysical properties, electrochemical properties and aromaticity (Wu et al., 2013; Guberman-Pfeffer et al., 2019; Penty et al., 2022). For example, the special annular structure of macrocycles is benefit for intermolecular contact and the transfer of electric charges in all directions. And in contrast to normal linear polymers, *p*-conjugated macrocycles lack end groups that could trap charge and degrade performance of devices (Ball et al., 2016) (Ball et al., 2019) (Ghosh et al., 2019). In addition, the *p*-conjugated macrocycles have tunable cavities and can be multifunctional by introducing building moieties with special features, which can be used as good candidates for novel supramolecular materials. Recently, many kinds of conjugated macrocycles with various shape have been extensively reported as specific hosts for supramolecular binding to other guests, such as fullerenes (Kawase et al., 2003; Lu et al., 2019; Jain et al., 2021; Wang et al., 2021). In addition to binding with other guests, conjugated macrocycles can also achieve self-assembly to construct macroscopic structures such as columnar 1D nanotubes, 2D porous networks, and 3D complexes benefiting from the non-foldable and fully *p*-conjugated backbones (Jung et al., 2006; Suzuki et al., 2010; He et al., 2013; Lee et al., 2016).

**FIGURE 1:**
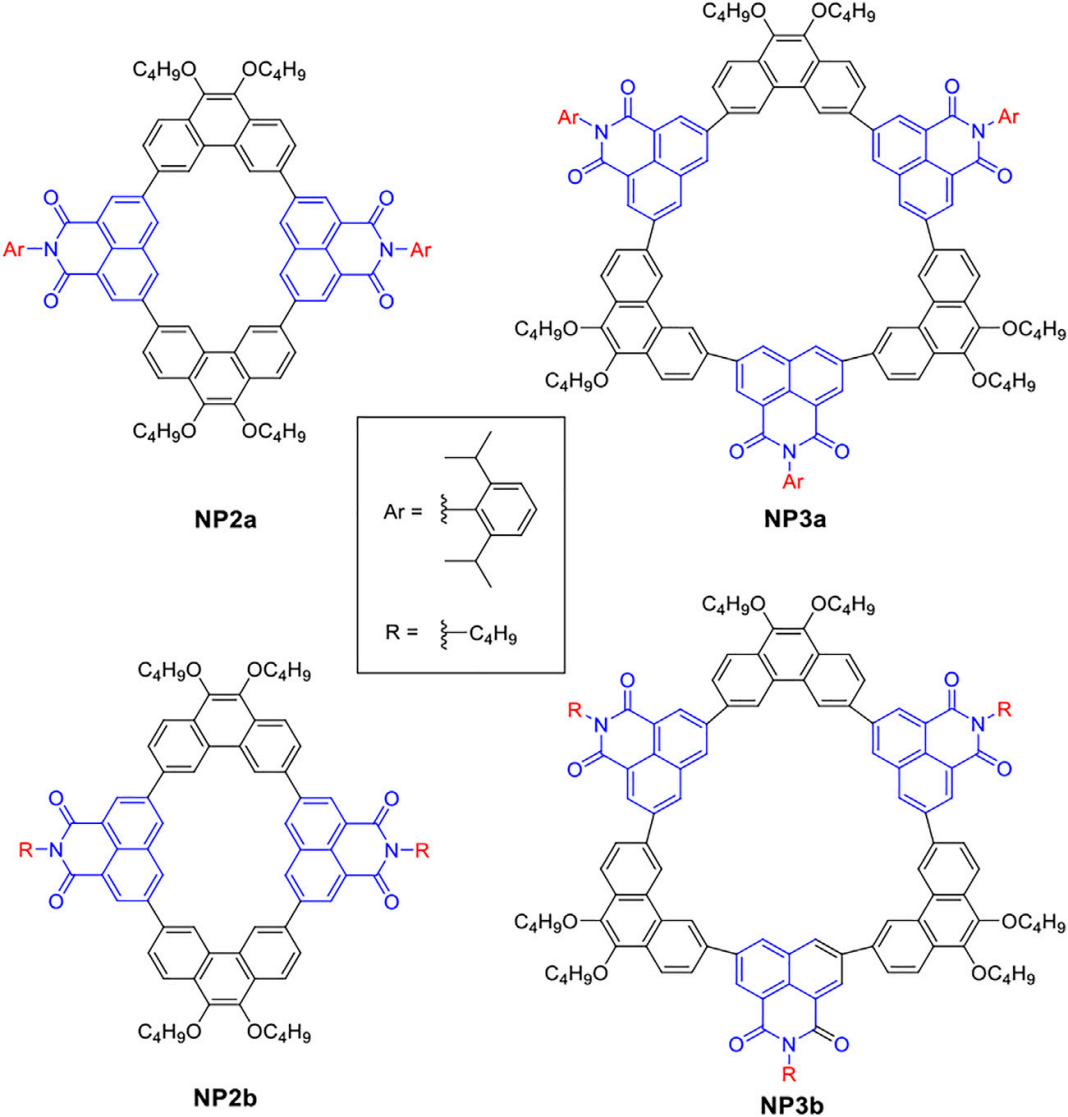
Four conjugated macrocycles based on phenanthrene (Phen) and 1, 8-naphthalimide (NMI).

However, the precise wet synthesis of macrocycles is still a challenging task and most of the conjugated macrocycles reported so far are composed of electron donors, such as the macrocyclic structures constructed by phenanthrene and benzene (Wang et al., 2021). The introduction of electron acceptors into macrocycles is beneficial to enhance the intramolecular charge transfer, which greatly affects their photophysical and electrochemical properties, as well as device performance and supramolecular assembly behavior. Naphthalimide derivatives are typical electron acceptor materials. Due to their excellent stability, easy functionalization, and tunable energy levels, they can be used to construct various organic optoelectronic devices wtih broad application prospects (Cheng et al., 2018; Genene et al., 2019; Lee et al., 2019; Chen et al., 2020). Among the derivatives of naphthalimide, 1,8-naphthalimide (NMI) can be used to construct conjugated macrocycles thanks to its easily functionalized position and suitable bond angle (Do et al., 2017; Boonnab et al., 2021). Herein, we utilize the electron acceptor 1,8-naphthalimide (NMI) and *p*-conjugated phenanthrene (Phen) as building blocks to construct a series of conjugated macrocycles with different sizes (Figure 1). Among them, the naphthalimide units of **NP2a** and **NP3a** are substituted with aryl groups, while those of **NP2b** and **NP3b** are substituted with alkyl groups. In this article, the synthetic method, molecular structure, photophysical properties, self-assembly behavior and supramolecular interactions with the typical guest molecule fullerene C_70_ of the four macrocycles will be systematically studied and reported.

## Synthesis

**SCHEME 1:**
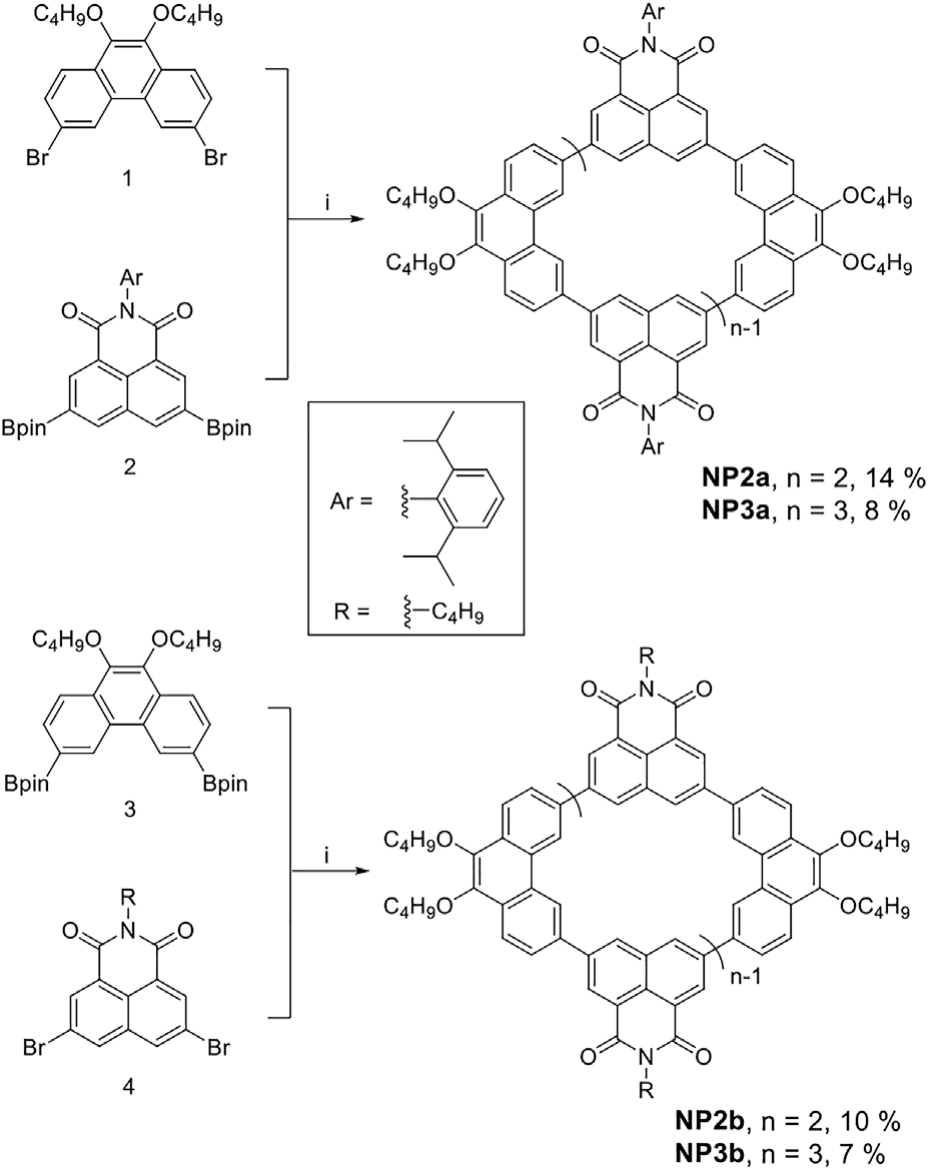
Synthesis of conjugated macrocycles NP2a, NP3a, NP2b and NP3b. i) Pd Xphos G2, K_3_PO_4_, THF/H_2_O, 60°C.

The synthetic routes of *p*-conjugated macrocycles are shown in Scheme 1. The precursor compounds one and two were both synthesized by literature methods (Phulwale et al., 2016); (Lu et al., 2018); (Xue et al., 2013), followed by Suzuki coupling reaction in dilute solution. The crude product was preliminarily separated by silica gel column and then futher purified by recycling preparative gel permeation chromatography (GPC) to obtain two sizes of macrocycles **NP2a** and **NP3a** with yields of 14% and 8% respectively.

Similar macrocycles **NP2b** and **NP3b** were also synthesized according to the same method, in which the substituent on 1,8-naphthalimide (NMI) was changed from aryl (in **NP2a** and **NP3a**) to alkyl to study the effect of substituent type on macrocyclic structure and physicochemical properties. It should be noted that the borylation reaction of compound 4 was difficult to perform and then an alternative cyclization route was adopted. The compound 3 was obtained by Miyaura borylation reaction, and then the Suzuki coupling of equimolar amounts of reactant three and four was carried out. **NP2b** and **NP3b** were obtained after purified by silica gel column and GPC with yields of 10% and 7%, respectively. The low yields of **NP2b** and **NP3b** may be due to the increased steric hindrance of cyclization after the borylationof phenanthrene units, which caused the reaction to produce a large amount of linear polymerization by-products. All newly synthesized compounds had been characterized and confirmed by ^1^H/^13^C NMR and high resolution mass spectrum (see SI).

### Ground-state geometry and theoretical calculations

**FIGURE 2:**
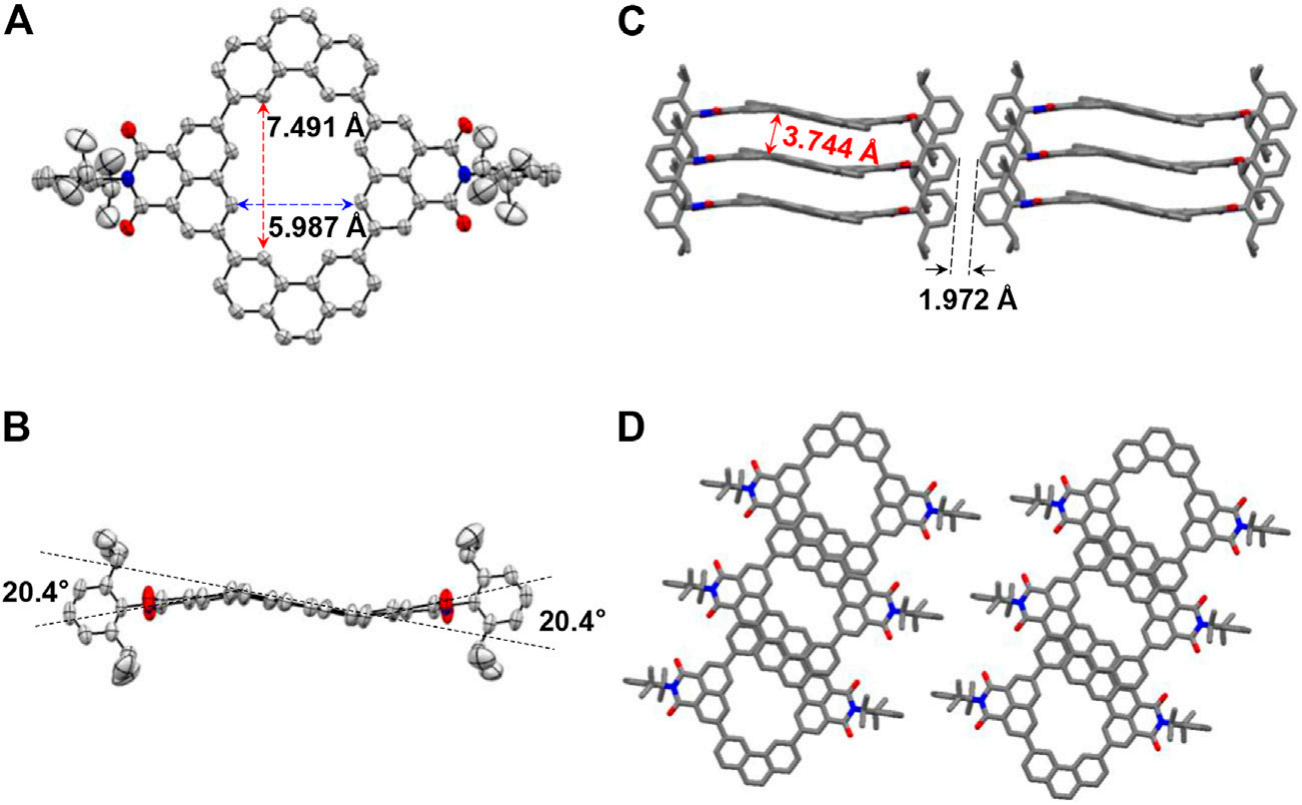
X-ray crystallographic structures of NP2a. **(A)** Top view and **(B)** side view of the molecule. **(C,D)** 3D packing structures of macrocycles.

In order to further confirm the structural characteristics of the macrocycles, single crystals of **NP2a** were obtained as yellow bulk crystals by slowly volatilizing in chloroform solution under ambient conditions, and single crystal X-ray diffraction analysis was performed. As is shown in Figure 2A, **NP2a** has a near-planar structure, in which the long axial length of the macrocycle is 7.491 Å while the short axial length is 5.987 Å. Taking the phenanthrene (Phen) units as the base plane, the two naphthalimide (NMI) building blocks are slightly twisted in opposite directions with a dihedral angle of 20.4°(Figure 2B). In addition, there are two parallel **NP2a** molecules in one unit cell and the long-range stacking of **NP2a** is layered with a *p*-π spacing of 3.744 Å (Figures 2C,D).

Many methods had also been tried to grow the single crystals of **NP3a**. Unfortunately, due to the twisted topology of the macrocycle, single crystals suitable for X-ray diffraction analysis were not obtained and the structure of **NP3a** was optimized by DFT calculations at B3LYP/6–31G (d,p) level of theory. As shown in Supplementary Figure S2, the macrocycle **NP3a** shows an extremely twisted geometry, and the dihedral angles between the phenanthrene (Phen) units and the naphthalimide (NMI) units vary between 50 and 55°. It can be inferred that the near-planar macrocycle **NP2a** constructed from building blocks with perfect bonding angles has a completely different topology from the macrocycle **NP3a** constructed with imperfect cyclization angles, which may be the reason for different physicochemical properties and assembly behaviors of the two macrocycles. When it comes to similar macrocycles **NP2b** and **NP3b**, we can find that they only have changes in substituents compared with **NP2a** and **NP3a**, which will not drastically affect the structure of their conjugated skeletons. The specific molecular geometries are also calculated by DFT calculations, where **NP2b** adopts a near-planar structure consistent with **NP2a**, and **NP3b** shows twisted ring structure similar to **NP3a** (Supplementary Figure S2).

**FIGURE 3:**
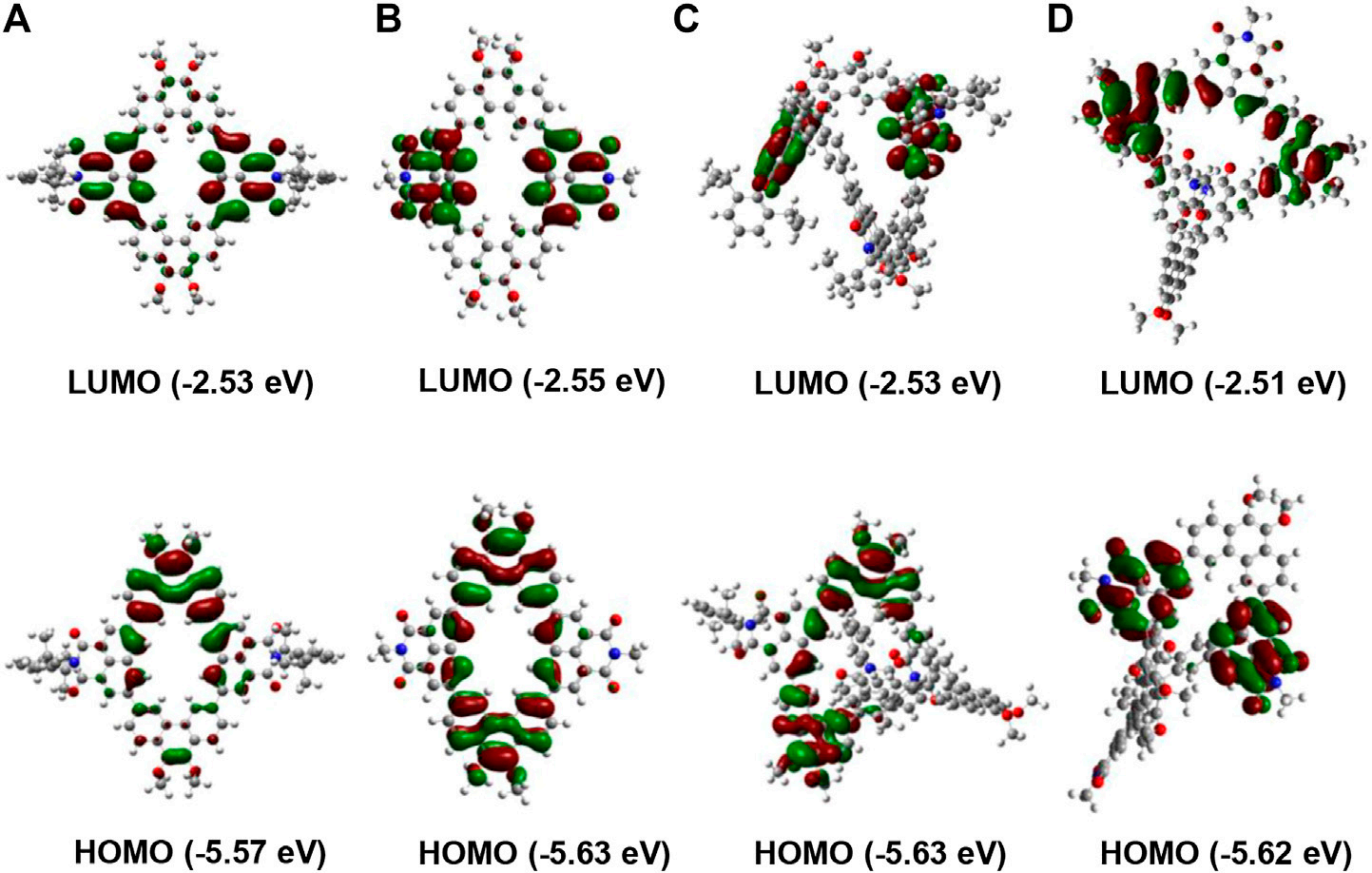
Frontier molecular orbital profiles and energy levels of the macrocycles calculated by DFT. **(A)** NP2a, **(B)** NP2b **(C)** NP3a, **(D)** NP3b.

To further understand the spatial geometry and electronic properties of these conjugated macrocycles, density functional theory (DFT) calculations based on first-principles were conducted. To simplify the calculation, all long alkyl groups in these macrocycles were replaced by methyl groups. As shown in Figure 3, the distribution of electron cloud in the alkyl-substituted molecules **NP2b**/**NP3b** is basically the same as that of the aryl-substituted macrocycles **NP2a**/**NP3a**. Both **NP2a** and **NP2b** show a planar structure, where the LUMOs are mainly distributed on two naphthalimide (NMI) building blocks while the HOMOs show main weight on two phenanthrene (Phen) units. On the other hand, the electron cloud distributions of **NP3a** and **NP3b** show great imbalance. The LUMOs are mainly localized on two of the naphthalimide (NMI) units while the other naphthalimide unit has almost no delocalization. Similarly, the HOMOs are distributed on two of the phenanthrene (Phen) units while the other phenanthrene (Phen) unit shows no HOMO electron cloud. These may be due to the overly distorted geometry of the macrocycles **NP3a** and **NP3b**, which greatly affects the conjugated backbone. In addition, the LUMO and HOMO levels of macrocycles can also be calculated (Shetty et al., 1996), in which the band gaps of the trimers **NP3a**/**NP3b** are all larger than the corresponding dimer **NP2a**/**NP2b**, which can also be attributed to the twisted molecular structure.

### Photophysical properties

**FIGURE 4:**
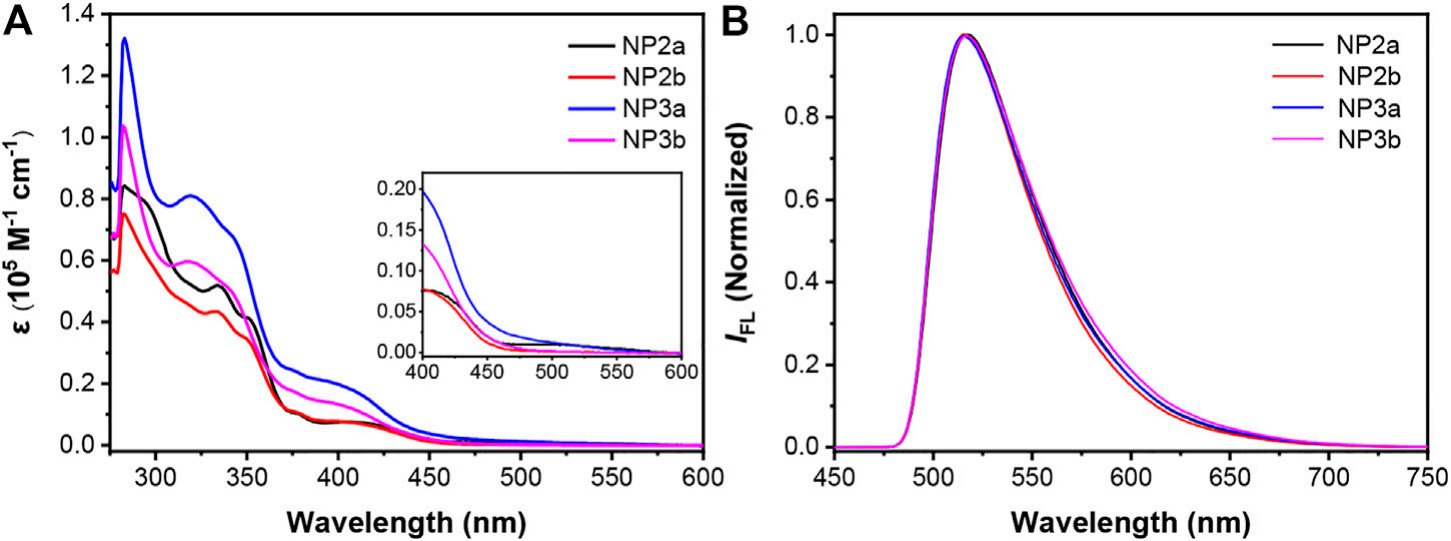
**(A)** UV−vis absorption and **(B)** normalized fluorescence spectra of NP2a, NP2b, NP3a and NP3b measured in toluene (1 × 10^–5^ M). Inset in **(A)** shows the magnified spectra at the long-wavelength region.

The newly synthesized macrocycles are all yellow solids with good solubility, which can be dissolved in most organic solvents, such as toluene, chloroform, dichloromethane and tetrahydrofuran. The absorption spectra and fluorescence spectra of **NP2a**, **NP3a**, **NP2b** and **NP3b** were measured in toluene (ca. 10^−5^ M). As shown in Figure 4A, there are two main absorption peaks in the absorption spectrum of each macrocycle, where the peak in the high-energy region corresponds to the *p*-π* transition of electrons in the conjugated backbone of macrocycle while the absorption peak in the low-energy region corresponds to the charge transfer from the electron-donor phenanthrene (Phen) units to the electron-acceptor naphthalimide (NMI) units. In addition, the molar extinction coefficients and optical band gaps of four macrocycles were calculated according to the absorption spectra, which are shown in Table 1.

**TABLE 1:** Photophysical Properties and Energy Levels of the macrocycles.

	ε[105 M−1cm−1]	λabs [nm]	λonset [nm]	λem [nm]	Egopt [eV]	Egcal [eV]
**NP2a**	0.85	283	459	517	2.70	3.04
**NP2b**	0.75	282	454	515	2.73	3.08
**NP3a**	1.04	283	445	516	2.79	3.10
**NP3b**	0.88	282	446	516	2.78	3.11

Comparing the conjugated macrocycles **NP2a** and **NP3a**, it is found that **NP3a** has a larger molar extinction coefficient than **NP2a**, which indicates that the conjugated system of **NP3a** has been expanded. However, the optical band gap of **NP3a** decreases compared to that of **NP2a**, which may be attributed to that the distorted structure of **NP3a** affects the continuity of the conjugated system, weakening the conjugation to a certain extent. The same phenomenon is also observed in alkyl-substituted macrocycles **NP2b** and **NP3b**. In addition, it is interesting to find that the aryl-substituted macrocycle **NP2a** have a larger molar extinction coefficient than the alkyl-substituted macrocycle **NP2b** although the trend of the absorption spectrum has no obvious change. This indicates that though the conjugated backbones of the two macrocycles have not changed significantly, the different substituents may affect the behavior of molecules in solution, resulting in different molar extinction coefficients. The above phenomenon also occurs in **NP3a** and **NP3b**.

Different from the absorption spectra, the fluorescence spectra of the four conjugated macrocycles in toluene solution are basically the same and the maximum emission wavelengths are all around 516 nm, which can be deduced that the influence of the substituents as well as the expansion of the conjugated system on the fluorescence emission is negligible for the four macrocycles (Figure 4B **)**. The absolute fluorescence quantum yields of **NP2a, NP3a, NP2b** and **NP3b** in solution were determined to be 30.8%, 28.85%, 25.9% and 25.57% respectively, by using the integrating sphere technique. The absolute fluorescence quantum yields of **NP2a, NP3a, NP2b** and **NP3b** in solid were also estimated as 5.17%, 9.79%, 1.40% and 2.62% relatively. The difference may be attributed to the solid phase stacking of the macrocycles.

In order to further investigate the intramolecular charge transfer (ICT) of the macrocycles, the solvatochromic effects on the absorption and PL features were investigated. It was found that both four kinds of п-conjugated macrocycles had similar solvatochromic effects (Supplementary Figures S3, S4). For example, although the absorption spectra of **NP2a** in different solvents showed negligible change, its fluorescence spectra showed remarkable solvation effect, indicating that there was significant donor-acceptor electron transfer in the macrocycle. And the more obvious solvation effect in fluorescence spectra than absorption spectrum may be due to that the excited electrons are more easily polarized (Zhu et al., 2018). In addition, cyclic voltammetry (CV) measurements of macrocycles **NP2a, NP3a, NP2b** and **NP3b** were also carried out in a solution of tetrabutylammonium hexafluorophosphate (n-Bu_4_NPF_6_) in anhydrous DCM with a scan rate of 50 mV s^−1^. As shown in Supplementary Figure S5, all the four macrocycles display similiar irreversible redox waves. From the onset potential of the first reductive wave, the LUMO energy levels of **NP2a, NP3a, NP2b** and **NP3b** are estimated as −3.02, -3.03, -3.05 and -3.02 eV, respectively.

### Self-assembly

**FIGURE 5:**
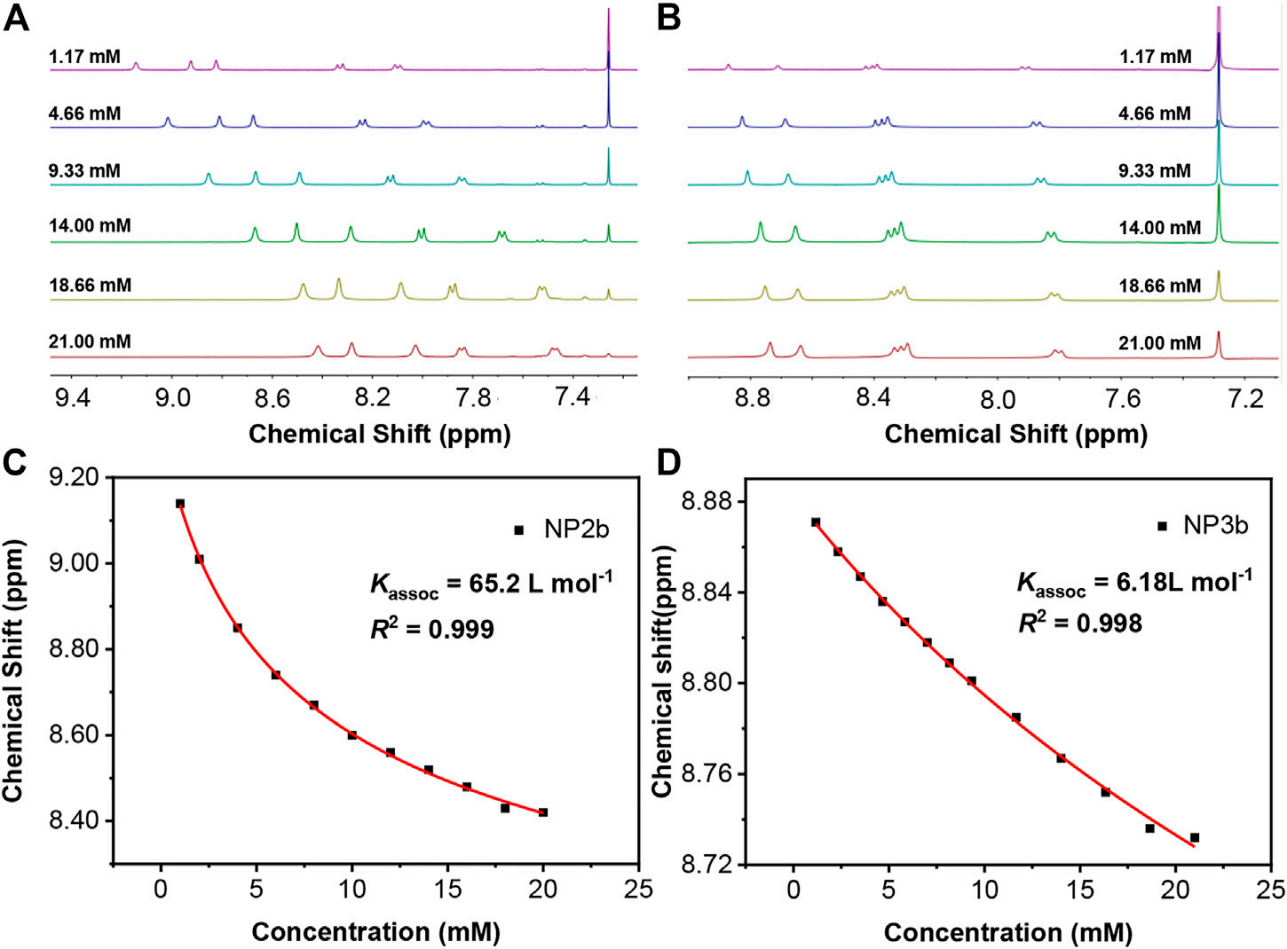
^1^H NMR (in CDCl_3_) spectroscopy at different concentrations for **(A)** NP2b **(B)** NP3b. Concentration-dependent images of chemical shifts fitted by a monomer-dimer model and self-assembly constants of **(C)** NP2b, **(D)** NP3b. K_assoc_ is the self-aggregating association constant and *R* ^2^ is the coefficient of determination.

It is found that the aromatic chemical shifts of **NP2b** and **NP3b** were dependent on concentration when ^1^H NMR spectroscopy was conducted. What’s more, the different absorption phenomena of macrocycles with different substituents also implied that there may be a special self-assembly phenomenon in solution. As is shown in Figure 5A, the chemical shift of aromatic proton in the lowest field of **NP2b** varied from δ = 9.14 to 8.42 ppm as the concentration changed from 1.13 to 21 mM. Similar phenomena was also been observed in **NP3b**, where the proton varied from 8.88 to 8.73 ppm in the same concentration range (Figure 5B). All of these suggest that the alkyl-substituted macrocycles self assemble with π–π stacking and the upfield shifts of the aromatic protons can be attributed to the influence of ring-current magnetic anisotropy of the adjacent macrocycle (Mao et al., 2020); (Shetty et al., 1996). In contract, the chemical shifts did not change significantly even when the concentration of aryl-substituted **NP2a**/**NP3a** was increased from 1.13 to 21 mM (Supplementary Figure S3), implying negligible self-aggregation, which may be due to the fact that the rigid aryl substituents greatly restrict the *p*-π stacking of macrocycles in solution.

Using the monomer-dimer model (Zhao and Moore, 2003) to simulate the chemical shift as a function of concentration, the self-association constants of **NP2b** and **NP3b** can be obtained. This model assumes that monomer-dimer equilibrium is the predominant process and higher order aggregations are negligible, which is sufficient for self-assembled systems in moderately polar chlorinated hydrocarbon solvents such as chloroform. The association constants for the self-aggregation of **NP2b** and **NP3b** were fitted by a computational method developed by Horman and Dreux (Horman and Dreux, 1984). As shown in Figure 5, the self-aggregating association constants of **NP2b** and **NP3b** are 65.2 and 6.18 L mol^−1^, respectively (Figures 5C,D). In addition, if macrocycles that can self-assemble in solution are applied to thin-film field-effect transistors, better performance may be obtained (Luo et al., 2010) (He et al., 2013), and these further studies are also underway in our laboratory.

### Binding behaviour with fullerenes

There is an inner cavity with a defined size in the macrocycle, which is beneficial for it to act as the host and combine with some guest molecules to achieve supramolecular binding. Here, C_70_ was selected as the typical guest molecule, and was added dropwise to the deuterated chloroform solutions of **NP2a**, **NP3a**, **NP2b** and **NP3b**, respectively. The mixture was tested by ^1^H NMR at room temperature and the supramolecular binding behavior was characterized by chemical shift.

**FIGURE 6:**
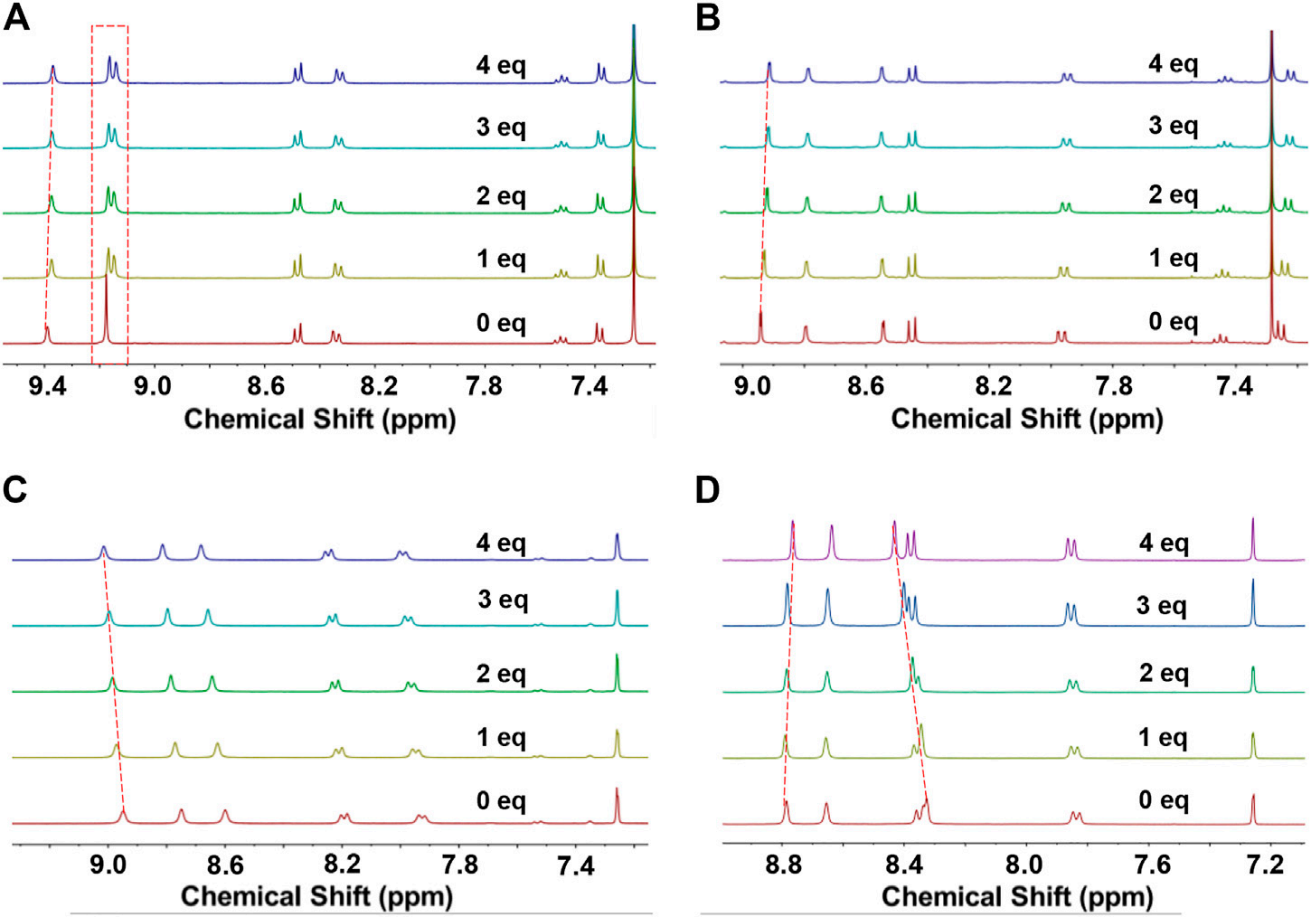
^1^H NMR spectra of different concentrations of C_70_ added to the CDCl_3_ solution of macrocycle. **(A)** NP2a, **(B)** NP3a **(C)** NP2b and **(D)** NP3b.

As shown in Figure 6A, when C_70_ was dropped into the solution of macrocycle **NP2a**, the two groups of aromatic protons in the macrocycle gradually separated from the original overlapping state. In addition to the obvious separation of protons, the aromatic protons in **NP2a** also showed a slight upfield shift trend with the addition of C_70_. All of these observations indicated that there was an obvious interaction between the macrocycle **NP2a** and C_70_, which significantly changed the chemical environment of the protons in the original solution of **NP2a**. Similar dimer macrocycle **NP2b** also showed changes, in which the aromatic proton shifted slightly to the downfield from δ = 8.96–9.01 ppm as the addition of C_70_ increased from 0 eq to 4eq (Figure 6C), demonstrating that there is also a weak supramolecular interaction between the host **NP2b** and guest C_70_. In contract, the trimer macrocycles **NP3a** and **NP3b** exhibited more pronounced chemical shift changes than dimer macrocycles **NP2a** and **NP2b** (Figures 6B,D). The chemical shift of the protons of **NP3a** and **NP3b** varied from 8.95 to 8.90 ppm and from 8.81 to 8.75 ppm, respectively, as the addition of C_70_ increased from 0 eq to 4eq.

**FIGURE 7:**
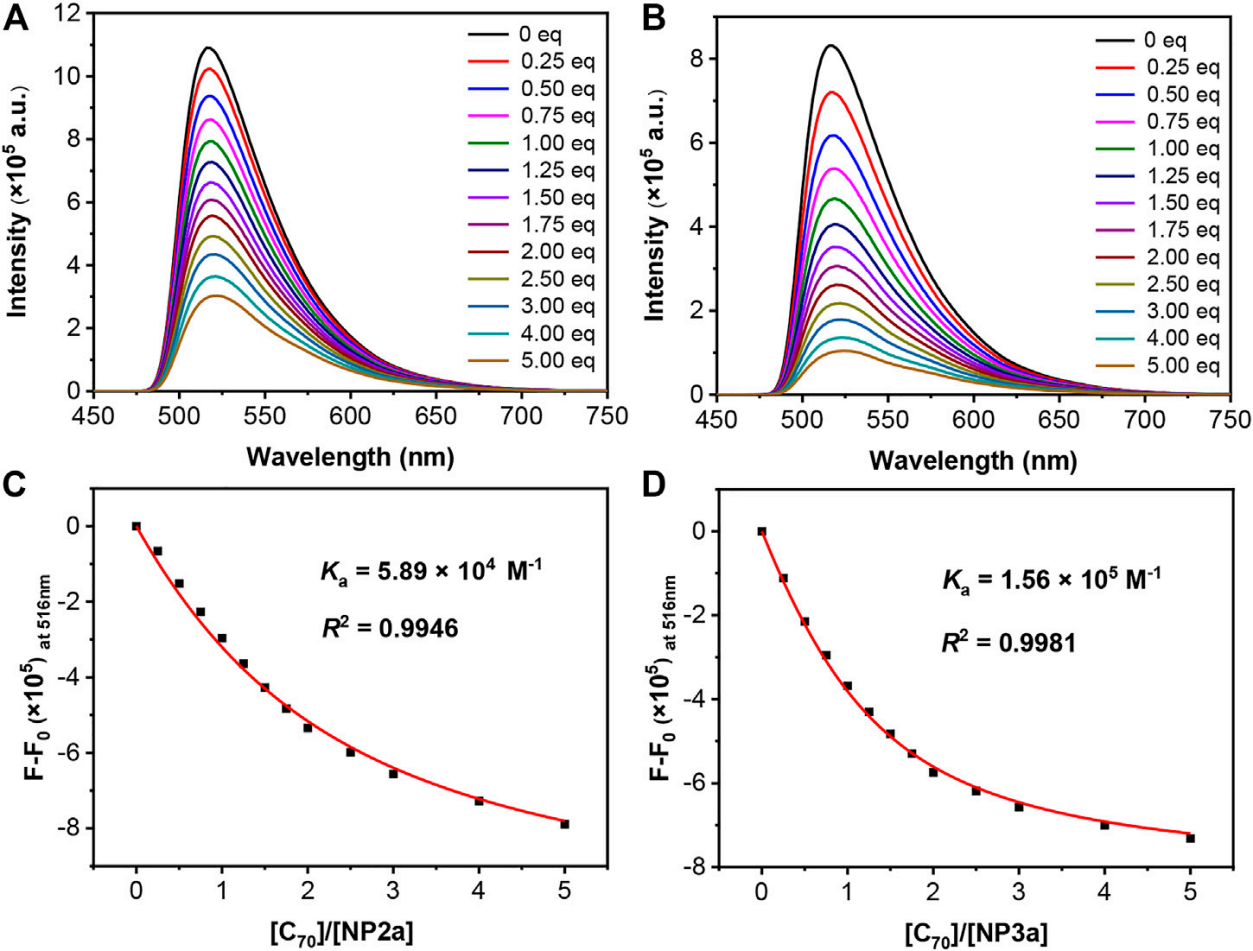
Fluorescence spectral change of **(A)** NP2a and **(B)** NP3a during titration with C_70_. Fitting curves on the relative fluorescent intensity of **(C)** NP2a, **(D)** NP3a for obtaining K_a_, *R* ^2^ is the coefficient of determination.

In order to quantitatively study the supramolecular assembly of macrocycles with fullerenes, C_70_ was added to the toluene solution of macrocycles **NP2a**, **NP3a**, **NP2b** and **NP3b** (ca. 10^−5^ M) respectively and the change of the fluorescence emission spectrum of the mixture was monitored. As shown in Figure 7, with the continuous addition of C_70_, the maximum emission peaks of the macrocycles **NP2a** and **NP3a** were all quenched to varying degrees, indicating that supramolecular complexes had been produced in solution. Similar fluorescence quenching was also observed in toluene solutions of **NP2b** and **NP3b** with the addition of C_70_ (Supplementary Figure S4). By a mole ratio plot based on fluorescence emission spectrum data (Supplementary Figure S7), a 1:1 stoichiometry was confirmed for **NP2a/NP3a/NP2b/NP3b** and C_70_ (Thordarson, 2011) (Brynn Hibbert and Thordarson, 2016) (Li et al., 2014). According to the non-linear curve-fitting method (Yang et al., 2020), the binding constants of **NP2a**, **NP2b**, **NP3a** and **NP3b** with C_70_ were calculated to be 5.89×10^4^ M^−1^, 2.68×10^4^ M^−1^, 1.56×10^5^ M^−1^ and 3.14×10^4^ M^−1^respectively, indicating that trimers **NP3a** and **NP3b** have stronger binding to C_70_ than dimers **NP2a** and **NP2b**, which is consistent with the results of the ^1^H NMR titration test. The stronger supramolecular assembly with C_70_ may be related to the larger internal cavity and non-planar topology of macrocycles **NP3a** and **NP3b**.

Considering that the macrocycles are consisting with electron-deficient naphthalimide units, the supramolecular assembly between **NP2a/NP3a/NP2b/NP3b** and the electron-rich guest pyrene was also investigated. The concentration of the macrocycles was kept constant, and the guest pyrene was added at room temperature according to the equivalent. The variation of characteristic peaks was monitored by NMR (Supplementary Figure S9, S10). The results show that only the planar macrocycles **NP2a** and **NP2b** are obviously encapsulated with pyrene, while the ^1^H NMR spectra of **NP3a** and **NP3b** changed little, indicating that they could not bind with pyrene. This may be due to the distorted geometry of **NP3a**/**NP3b** which is not suitable for binding with planar molecules. By a mole ratio plot based on chemical shift data, a 1:1 stoichiometry was confirmed for **NP2a/NP2b** and pyrene (Zeng et al., 2022). Through non-linear fitting, the binding constants of **NP2a/NP2b** with pyrene are 327.43 and 253.33 M^−1^ respectively.

## Conclusion

In summary, four macrocycles **NP2a**, **NP3a**, **NP2b** and **NP3b** based on phenanthrene (Phen) and naphthalimide (NMI) have been successfully synthesized. Their structures were confirmed by ^1^H NMR, ^13^C NMR, single crystal X-ray analysis and high-resolution mass spectrometry, in which the dimer macrocycles **NP2a** and **NP2b** adopt near-plane structure while the trimer **NP3a** and **NP3b** are twisted ring structure. Both DFT theoretical calculations and UV-Vis absorption spectra indicate that **NP3a** and **NP3b** have larger band gap than the corresponding dimer macrocycles **NP2a** and **NP2b**, which may be due to the distorted topology of the trimer macrocycles. In addition, we also found that the alkyl-substituted macrocycles **NP2b** and **NP3b** exhibited obvious self-aggregation behavior in solution, while the aryl-substituted macrocycles **NP2a** and **NP3a** had no similar phenomenon, which suggests that the alkyl-substituted macrocycles are more favorable for the formation of tight *p*-π stacking than the aryl-substituted macrocycles. Finally, ^1^H NMR titration and fluorescence emission spectrometry showed that there were supramolecular interactions between the four conjugated macrocycles and fullerene C_70_, and the binding between **NP3a**/**NP3b** and C_70_ is stronger than that in **NP2a**/**NP2b**. This work demonstrates the potential of macrocycles containing special electron acceptors NMI for the development of supramolecular chemistry.

## Data Availability

The original contributions presented in the study are included in the article/Supplementary Material, further inquiries can be directed to the corresponding authors.

## References

[B1] Ball M. Zhang B. Zhong Y. Fowler B. Xiao S. Ng F. (2019). Conjugated macrocycles in organic electronics. Acc. Chem. Res. 52, 1068–1078. 10.1021/acs.accounts.9b00017 30869865

[B2] Ball M. Zhong Y. Fowler B. Zhang B. Li P. Etkin G. (2016). Macrocyclization in the design of organic n-type electronic materials. J. Am. Chem. Soc. 138 (39), 12861–12867. 10.1021/jacs.6b05474 27666433

[B3] Boonnab S. Chaiwai C. Nalaoh P. Manyum T. Namuangruk S. Chitpakdee C. (2021). Synthesis, characterization, and physical properties of pyrene-naphthalimide derivatives as emissive materials for electroluminescent devices. Eur. J. Org. Chem. 17, 2402–2410. 10.1002/ejoc.202100134

[B4] Brynn Hibbert D. Thordarson P. (2016). The death of the Job plot, transparency, open science and online tools, uncertainty estimation methods and other developments in supramolecular chemistry data analysis. Chem. Commun. 52, 12792–12805. 10.1039/C6CC03888C 27779264

[B5] Chen J. Zhuang X. Huang W. Su M. Feng L. W. Swick S. M. (2020). π-Extended naphthalene diimide derivatives for n-Type semiconducting polymers. Chem. Mat. 32, 5317–5326. 10.1021/acs.chemmater.0c01397

[B6] Cheng P. Li G. Zhan X. Yang Y. (2018). Next-generation organic photovoltaics based on non-fullerene acceptors. Nat. Photonics 12, 131–142. 10.1038/s41566-018-0104-9

[B7] Dey K. Mohata S. Banerjee R. (2021). Covalent organic frameworks and supramolecular nano-synthesis. ACS Nano 15, 12723–12740. 10.1021/acsnano.1c05194

[B8] Do T. T. Pham H. D. Manzhos S. Bell J. M. Sonar P. (2017). Molecular engineering strategy for high efficiency fullerene-free organic solar cells using conjugated 1, 8-naphthalimide and fluorenone building blocks. ACS Appl. Mater. Interfaces 9, 16967–16976. 10.1021/acsami.6b16395 28467709

[B9] Dou X. Pisula W. Wu J. Bodwell G. J. Müllen K. (2008). Reinforced self-assembly of hexa-peri-hexabenzocoronenes by hydrogen bonds: From microscopic aggregates to macroscopic fluorescent organogels. Chem. Eur. J. 14, 240–249. 10.1002/chem.200700921 17918756

[B10] Genene Z. Mammo W. Wang E. Andersson M. R. (2019). Recent advances in n-type polymers for all-polymer solar cells. Adv. Mat. 31, 1807275. 10.1002/adma.201807275 30790384

[B11] Ghosh S. Das S. Saeki A. Praveen V. K. Seki S. Ajayaghosh A. (2018). A hybrid organogel of a low band gap diketopyrrolopyrrole with PC71BM: Phase separated morphology and enhanced photoconductivity. ChemNanoMat 4, 831–836. 10.1002/cnma.201800149

[B12] Ghosh S. Prasanthkumar S. Das S. Saeki A. Seki S. Ajayaghosh A. (2022). Structurally directed thienylenevinylene self-assembly for improved charge carrier mobility: 2D sheets vs. 1D fibers. Chem. Commun. 58, 6837–6840. 10.1039/D2CC02111K 35616190

[B13] Ghosh S. Tsutsui Y. Suzuki K. Kaji H. Honjo K. Uemura T. (2019). Impact of the position of the imine linker on the optoelectronic performance of π-conjugated organic frameworks. Mol. Syst. Des. Eng. 4, 325–331. 10.1039/C8ME00079D

[B14] He Z. Xu X. Zheng X. Ming T. Miao Q. (2013). Conjugated macrocycles of phenanthrene: A new segment of [6, 6]-carbon nanotube and solution-processed organic semiconductors. Chem. Sci. 4, 4525–4531. 10.1039/C3SC52077C

[B15] Helmich F. Lee C. C. Nieuwenhuizen M. M. L. Gielen J. C. Christianen P. C. M. Larsen A. (2010). Dilution-induced self-assembly of porphyrin aggregates: A consequence of coupled equilibria. Angew. Chem. Int. Ed. 49, 3939–3942. 10.1002/anie.201000162 20379987

[B16] Horman I. Dreux B. (1984). Estimation of dimerisation constants from complexatin-induced displacements of ^1^H-NMR chemical shifts: Dimerisation of caffeine. Helvetica Chim. Acta 67, 754–764. 10.1002/hlca.19840670316

[B17] Hou J. L. Yi H. P. Shao X. B. Li C. Wu Z. Q. Jiang X. K. (2006). Helicity induction in hydrogen-bonding-driven zinc porphyrin foldamers by chiral C60-incorporating histidines. Angew. Chem. Int. Ed. 45, 796–800. 10.1002/anie.200502465 16355424

[B18] Jain K. Duvva N. Roy T. K. Giribabu L. Chitta R. (2021). Porphyrin bearing phenothiazine pincers as hosts for fullerene binding via concave–convex complementarity: Synthesis and complexation study. New J. Chem. 45, 19691–19703. 10.1039/D1NJ03727G

[B19] Jung S. H. Pisula W. Rouhanipour A. Räder H. J. Jacob J. Müllen K. (2006). A conjugated polycarbazole ring around a porphyrin. Angew. Chem. Int. Ed. 45, 4685–4690. 10.1002/anie.200601131 16789038

[B20] Kawase T. Tanaka K. Fujiwara N. Darabi H. R. Oda M. (2003). Complexation of a carbon nanoring with fullerenes. Angew. Chem. Int. Ed. 42, 1624–1628. 10.1002/anie.200250728 12698460

[B21] Kim H. G. Shin H. Ha Y. H. Kim R. Kwon S. K. Kim Y. H. (2019). Triplet harvesting by a fluorescent emitter using a phosphorescent sensitizer for blue organic-light-emitting diodes. ACS Appl. Mat. Interfaces. 11, 26–30. 10.1021/acsami.8b17957 30543096

[B22] Kudernac T. Lei S. Elemans J. A. A. W. De Feyter S. (2009). Two-dimensional supramolecular self-assembly: Nanoporous networks on surfaces. Chem. Soc. Rev. 38, 402–421. 10.1039/B708902N 19169457

[B23] Kumar S. Koo Y. H. Higashino T. Matsuda W. Ghosh S. Tsutsui Y. (2022). Truxenone triimide: Two-dimensional molecular arrangements of triangular molecules for air stable n-type semiconductors. Adv. Electron. Mat. 8, 2101390. 10.1002/aelm.202101390

[B24] Lee C. Lee S. Kim G. U. Lee W. Kim B. J. (2019). Recent advances, design guidelines, and prospects of all-polymer solar cells. Chem. Rev. 119, 8028–8086. 10.1021/acs.chemrev.9b00044 31181904

[B25] Lee S. Hirsch B. E. Liu Y. Dobscha J. R. Burke D. W. Tait S. L. (2016). Multifunctional tricarbazolo triazolophane macrocycles: One-pot preparation, anion binding, and hierarchical self-organization of multilayers. Chem.Eur. J. 22, 560–569. 10.1002/chem.201503161 26593327

[B26] Li J. Wang J. Li H. Song N. Wang D. Tang B. Z. (2020). Supramolecular materials based on AIE luminogens (AIEgens): Construction and applications. Chem. Soc. Rev. 49, 1144–1172. 10.1039/C9CS00495E 31971181

[B27] Li Z. Yang J. Yu G. He J. Abliz Z. Huang F. (2014). Synthesis of a water-soluble pillar[9]arene and its pH-responsive binding to paraquat. Chem. Commun. 50, 2841–2843. 10.1039/C3CC49535C 24501738

[B28] Loh K. P. Tong S. W. Wu J. (2016). Graphene and graphene-like molecules: Prospects in solar cells. J. Am. Chem. Soc. 138, 1095–1102. 10.1021/jacs.5b10917 26741946

[B29] Lu X. Gopalakrishna T. Y. Han Y. Ni Y. Zou Y. Wu J. (2019). Bowl-shaped carbon nanobelts showing size-dependent properties and selective encapsulation of C70. J. Am. Chem. Soc. 141, 5934–5941. 10.1021/jacs.9b00683 30905147

[B30] Lu X. Gopalakrishna T. Y. Phan H. Herng T. S. Jiang Q. Liu C. (2018). Global aromaticity in macrocyclic cyclopenta-fused tetraphenanthrenylene tetraradicaloid and its charged species. Angew. Chem. Int. Ed. 57, 13052–13056. 10.1002/anie.201807185 30067299

[B31] Luo J. Yan Q. Zhou Y. Li T. Zhu N. Bai C. (2010). A photoswitch based on self-assembled single microwire of a phenyleneethynylene macrocycle. Chem. Commun. 46, 5725–5727. 10.1039/C0CC00739K 20593084

[B32] Mao L. Hu Y. Tu Q. Jiang W. L. Zhao X. L. Wang W. (2020). Highly efficient synthesis of non-planar macrocycles possessing intriguing self-assembling behaviors and ethene/ethyne capture properties. Nat. Commun. 11, 5806. 10.1038/s41467-020-19677-x 33199747 PMC7669899

[B33] Ogoshi T. Yamagishi T. A. Nakamoto Y. (2016). Pillar-shaped macrocyclic hosts pillar[n]arenes: New key players for supramolecular chemistry. Chem. Rev. 116, 7937–8002. 10.1021/acs.chemrev.5b00765 27337002

[B34] Park C. H. Simmons H. E. (1968). Macrobicyclic amines. III. encapsulation of halide ions by in,in-1,(k + 2)-diazabicyclo[k.l.m.]alkane ammonium ions. J. Am. Chem. Soc. 90, 2431–2432. 10.1021/ja01011a047

[B35] Penty S. E. Zwijnenburg M. A. Orton G. R. F. Stachelek P. Pal R. Xie Y. (2022). The pink box: Exclusive homochiral aromatic stacking in a bis-perylene diimide macrocycle. J. Am. Chem. Soc. 144, 12290–12298. 10.1021/jacs.2c03531 35763425 PMC9348826

[B36] Phulwale B. V. Mishra S. K. Nečas M. Mazal C. (2016). Phenanthrylene-butadiynylene and phenanthrylene-thienylene macrocycles: Synthesis, structure, and properties. J. Org. Chem. 81, 6244–6252. 10.1021/acs.joc.6b00814 27398717

[B37] Ringsdorf H. Simon J. (1994). Snap-together vesicles. Nature 371, 284. 10.1038/371284a0 8090195

[B38] Savage N. (2011). Electronics: Organic growth. Nature 479, 557–559. 10.1038/nj7374-557a 22121517

[B39] Shetty A. S. Zhang J. Moore J. S. (1996). Aromatic π-stacking in solution as revealed through the aggregation of phenylacetylene macrocycles. J. Am. Chem. Soc. 118, 1019–1027. 10.1021/ja9528893

[B40] Song Q. Cheng Z. Kariuki M. Hall S. C. L. Hill S. K. Rho J. Y. (2021). Molecular self-assembly and supramolecular chemistry of cyclic peptides. Chem. Rev. 121, 13936–13995. 10.1021/acs.chemrev.0c01291 33938738 PMC8824434

[B41] Suzuki M. Comito A. Khan S. I. Rubin Y. (2010). Nanochannel array within a multilayered network of a planarized dehydro[24]annulene. Org. Lett. 12, 2346–2349. 10.1021/ol1006967 20402478

[B42] Thordarson P. (2011). Determining association constants from titration experiments in supramolecular chemistry. Chem. Soc. Rev. 40, 1305–1323. 10.1039/C0CS00062K 21125111

[B43] Wang J. Ju Y. Y. Low K. H. Tan Y. Z. Liu J. (2021). A molecular transformer: A π-conjugated macrocycle as an adaptable host. Angew. Chem. Int. Ed. 60, 11814–11818. 10.1002/anie.202102637 33751785

[B44] Whitesides G. M. Grzybowski B. (2002). Self-assembly at all scales. Science 295, 2418–2421. 10.1126/science.1070821 11923529

[B45] Wolffs M. Hoeben F. J. M. Beckers E. H. A. Schenning A. P. H. J. Meijer E. W. (2005). Sequential energy and electron transfer in aggregates of tetrakis[oligo(p-phenylene vinylene)] porphyrins and C60 in water. J. Am. Chem. Soc. 127, 13484–13485. 10.1021/ja054406t 16190697

[B46] Wu J. I. Fernández I. Schleyer P. V. R. (2013). Description of aromaticity in porphyrinoids. J. Am. Chem. Soc. 135, 315–321. 10.1021/ja309434t 23205604

[B47] Würthner F. Bauer C. Stepanenko V. Yagai S. (2008). A black perylene bisimide super gelator with an unexpected J-type absorption band. Adv. Mat. 20, 1695–1698. 10.1002/adma.200702935

[B48] Xue J. Y. Nakanishi W. Tanimoto D. Hara D. Nakamura Y. Isobe H. (2013). Convergent synthesis of hexameric naphthylene macrocycles with dicarboxylic imide appendages. Tetrahedron Lett. 54, 4963–4965. 10.1016/j.tetlet.2013.07.025

[B49] Yang L. Tan X. Wang Z. Zhang X. (2015). Supramolecular polymers: Historical development, preparation, characterization, and functions. Chem. Rev. 115, 7196–7239. 10.1021/cr500633b 25768045

[B50] Yang L. Zhang N. Han Y. Zou Y. Qiao Y. Chang D. (2020). A sulfur-containing hetero-octulene: Synthesis, host–guest properties, and transistor applications. Chem. Commun. 56, 9990–9993. 10.1039/D0CC04289G 32724946

[B51] Yang X. Lu R. Xue P. Li B. Xu D. Xu T. (2008). Carbazole-based organogel as a scaffold to construct energy transfer arrays with controllable fluorescence emission. Langmuir 24, 13730–13735. 10.1021/la8027226 18980355

[B52] Zeng F. Cheng L. Ou G.-C. Tang L.-L. Ding M.-H. (2022). Pyromellitic diimide-extended pillar[6]arene: Synthesis, structure, and its complexation with polycyclic aromatic hydrocarbons. J. Org. Chem. 87 (5), 3863–3867. 10.1021/acs.joc.1c03096 35171603

[B53] Zhao D. Moore J. S. (2003). Shape-persistent arylene ethynylene macrocycles: Syntheses and supramolecular chemistry. Chem. Commun. 2003, 807–818. 10.1039/B207442G 12739627

[B54] Zhou Y. Yan D. (2009). Supramolecular self-assembly of amphiphilic hyperbranched polymers at all scales and dimensions: Progress, characteristics and perspectives. Chem. Commun., 1172–1188. 10.1039/b814560c 19240868

[B55] Zhu G. Zhang Y. Hu Y. Zhao X. Yuan Z. Chen Y. (2018). Conjugated polymers based on 1, 8-naphthalene monoimide with high electron mobility. J. Polym. Sci. Part A Polym. Chem. 56, 276–281. 10.1002/pola.28891

