# Supplementary material for: Naphthalimide-based conjugated macrocycles possessing tunable self-assembly and supramolecular binding behaviours
Source: Front Chem. 2022 Dec 20;10:1094828. doi: 10.3389/fchem.2022.1094828 (PMC9807915; doi:10.3389/fchem.2022.1094828)
Supplement: Supplementary file 1 [file DataSheet1.pdf]

Supporting Information for

Naphthalimide-based conjugated macrocycles possessing tunable self-assembly and supramolecular binding properties

Table of Contents

1. Experimental section 1
  1.1 Materials and characterizations 1
  1.2 Synthesis 2
2. X-ray crystallographic data 4
3. DFT calculations 6
4. Additional Figures 7
5. ^{1}H, ^{13}C NMR and high resolution mass spectrometry 15
6. References 21

1. Experimental section

1.1. Materials and characterizations

All chemicals and reagents were purchased from commercial sources and used as received unless specified. The precursor compounds 3,6-dibromo-9,10-dibutoxy-phenanthrene (Phulwale et al., 2016); (Lu et al., 2018), 2-(2,6-diisopropylphenyl)-5,8-bis(4,4,5,5-tetramethyl-1,3,2-dioxaboro-lan-2-yl)-1*H*-benzo[*de*]isoquinoline-1,3(2*H*)-dione (Xue et al., 2013), 2,2'-(9,10-dibutoxyphenanthrene-3,6-diyl)bis(4,4,5,5-tetramethyl-1,3,2-dioxaborolane) (Gregolińska et al., 2018) and 5,8-dibromo-2-butyl-1*H*-benzo[*de*]isoquinoline-1,3(2*H*)-dione (Kiyotaki et al., 2020) were both synthesized following previously reported methods. Anhydrous tetrahydrofuran (THF) was distilled from sodium benzophenone ketyl. Dichloromethane (DCM) and chloroform were distilled from CaH_2. All reactions and manipulations were carried out with the use of standard inert atmosphere and Schlenk techniques. The ^{1}H NMR and ^{13}C NMR spectra were recorded in solution of $CDCl_3$ on Bruker DPX300/DPX400/DPX500 NMR spectrometer with tetramethylsilane (TMS) as the internal standard. The following abbreviations were used to explain the multiplicities: s (singlet), d (doublet), t (triplet), m (multiplet). Ultraviolet-visible-near infrared (UV-Vis-NIR) absorption spectra were recorded on a Perkin Elmer Lambda-750 spectrophotometer. Photoluminescence (PL) spectra were measured on an Edinburgh fluorescence spectrometer (FLS1000). The electrochemical measurements were carried out in anhydrous DCM with 0.1 M tetrabutylammonium hexafluorophosphate (n-Bu_4NPF_6) as the supporting electrolyte at room temperature under the protection of nitrogen. A glass carbon disk was used as working electrode, a platinum wire was used as counting electrode, and Ag/Ag^+ electrode as reference electrode. Redox couple ferrocenium/ferrocene was used as an internal standard.

1.2. Synthesis

Synthesis of NP2a and NP3a.

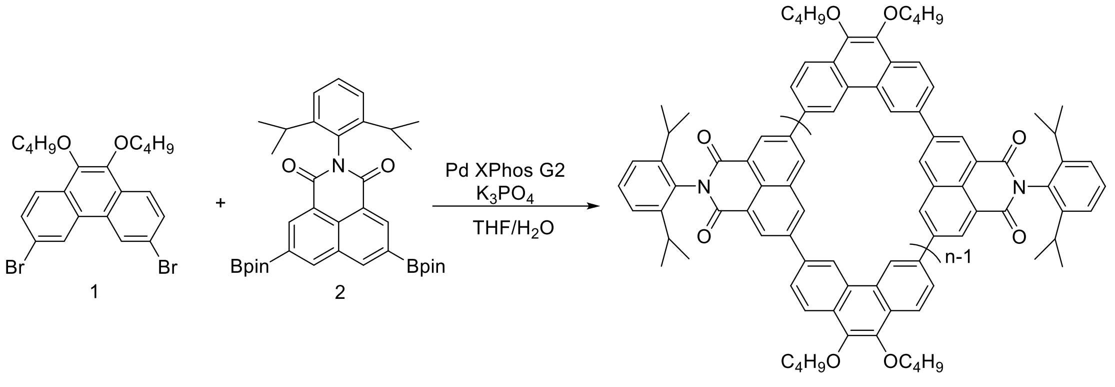

Compound 1 (221 mg, 0.46 mmol), 2 (280 mg, 0.46 mmol), XPhos Pd G2 (35 mg, 0.045 mmol), THF (150 mL) was added to a three-necked flask. The reaction system was carefully degassed and protected by nitrogen atmosphere, then K_3PO_4 solution (19.2 g dissolved in 150 mL H_2O) was injected to the mixture. The mixture was heated to 50 °C and stirred for 24 h. After the reaction, the organic solvent was removed under reduced pressure and then extracted with dichloromethane. After remove the solvent and dried over anhydrous sodium sulfate, the crude product was first purified by silica gel column chromatography (DCM: Hexane: ethyl acetate = 10: 5: 1), which can remove catalysts effectively. Then the mixture was further purified by preparative GPC using chloroform as solvent at a rate of 16 mL/min. The yellow solid macrocycle **NP2a** was obtained in 14 % yield (44 mg). ^{1}H NMR (400 MHz, $CDCl_3$, δ ppm): 9.39 (s, 4H), 9.18 (s, 8H), 8.48 (d, J = 8.5 Hz, 4H), 8.34 (d, J = 8.5 Hz, 4H), 7.52 (d, J = 7.8 Hz, 2H), 7.38 (d, J = 7.8 Hz, 8H), 4.34 (t, J = 6.6 Hz, 8H), 2.92 -2.77 (m, 4H), 2.05 -1.92 (m, 8H), 1.67 (dd, J = 15.1, 7.5 Hz, 8H), 1.21 (d, J = 6.8 Hz, 24H), 1.07 (t, J = 7.4 Hz, 12H). ^{13}C NMR (101 MHz, $CDCl_3$, δ ppm): 164.28, 145.71, 143.95, 140.25, 135.85, 133.49, 132.03, 130.93, 130.47, 130.25, 129.64, 128.95, 127.45, 125.41, 124.14, 123.87, 123.43, 122.02, 77.34, 77.02, 76.71, 73.65, 32.62, 29.26, 24.07, 19.54, 14.07. HRMS (ESI, m/z): [(M+H)+] calcd for $C_{92}H_{93}N_2O_8$, 1351.67699; found, 1351.67699. The yellow solid macrocycle **NP3a** was obtained in 8 % yield (40 mg). ^{1}H NMR (400 MHz, $CDCl_3$, δ ppm): 8.95 (d, J = 1.5 Hz, 6H), 8.80 (s, 6H), 8.54 (s, 6H), 8.45 (d, J = 8.5 Hz, 6H), 7.97 (d, J = 8.5 Hz, 6H), 7.45 (t, J = 7.7 Hz, 3H), 7.27-7.23 (m, 6H), 4.36 (t, J = 6.6 Hz, 12H), 2.74 (dt, J = 13.5, 6.7 Hz, 6H), 2.04-1.97 (m, 12H), 1.68 (dt, J = 14.8, 7.4 Hz, 12H), 1.14-1.08 (m, 18H), 1.04 (d, J = 6.8 Hz, 36H). ^{13}C NMR (101 MHz, $CDCl_3$,

δ ppm): 164.23, 145.59, 143.59, 142.01, 137.73, 133.02, 132.53, 129.77, 128.65, 124.02, 122.86, 77.34, 77.03, 76.71, 73.64, 32.63, 29.07, 23.91, 19.54, 14.08. HRMS (ESI, m/z): [(M+H)+] calcd for $C_{138}H_{136}N_3O_{12}$, 2027.01185; found, 2027.00867.

Synthesis of NP2b and NP3b.

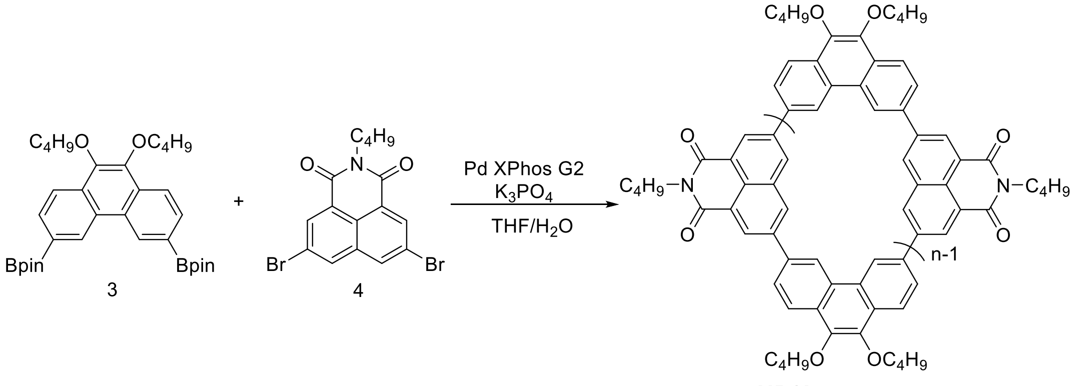

Macrocycles **NP2b** and **NP3b** were synthesized similarly as described for synthesis of **NP2a** and **NP3a**. The yellow solid macrocycle **NP2b** was obtained in 10 % yield (28 mg). ^{1}H NMR (400 MHz, $CDCl_3$, δ ppm):8.99 (s, 4H), 8.78 (s, 4H), 8.64 (s, 4H), 8.21 (s, 4H), 7.97 (s, 4H), 4.28 (s, 8H), 4.22 (s, 4H), 2.03 -1.93 (m, 8H), 1.77 (d, J = 8.2 Hz, 4H), 1.73- 1.66 (m, 8H), 1.52 (d, J = 7.3 Hz, 4H), 1.13 (t, J = 7.3 Hz, 12H), 1.04 (t, J = 7.3 Hz, 6H). ^{13}C NMR (101 MHz, $CDCl_3$, δ ppm): 163.31, 143.67, 134.74, 133.96, 129.65, 129.38，129.21, 129.19，128.97, 128.95，128.73, 128.67，128.20, 128.05, 127.80, 126.03, 125.79, 125.54, 120.68, 73.26, 32.99, 30.50, 29.98, 20.63, 19.85, 14.21, 13.92. HRMS (ESI, m/z): [(M+H)+] calcd for $C_{76}H_{75}N_2O_8$, 1143.55179; found, 1143.55179. The yellow solid macrocycle **NP3b** was obtained in 7 % yield (20 mg). ^{1}H NMR (400 MHz, $CDCl_3$, δ ppm): 8.84 (s, 6H), 8.68 (s, 6H), 8.39 (d, J = 8.6 Hz, 6H), 8.37 (s, 6H), 7.88 (d, J = 8.4 Hz, 6H), 4.31 (t, J = 6.6 Hz, 12H), 4.15 (s, 6H), 1.95 (dd, J = 14.6, 7.1 Hz, 12H), 1.70-1.64 (m, 6H), 1.63 (s, 12H), 1.36 (d, J = 7.8 Hz, 6H), 1.07 (d, J = 7.4 Hz, 18H), 0.82 (t, J = 7.2 Hz, 9H). ^{13}C NMR (101 MHz, $CDCl_3$, δ ppm): 163.93, 143.49, 141.54, 137.56, 132.63, 132.19, 130.76 , 129.61, 128.52, 126.73, 126.04, 123.14, 122.64, 229.44，29.52, 77.28, 77.02 (s, 16H), 76.70, 73.51, 24.88, 20.23, 19.56 , 14.08, 13.64. HRMS (ESI, m/z): [(M+H)+] calcd for $C_{114}H_{112}N_3O_{12}$, 1714.82405; found, 1714.82405.

2. X-ray crystallographic data

Table S1. X-ray crystal data and structure refinement details for **NP2a**.

	**NP2a**
formula	$C_{98}H_{96}Cl_{18}N_2O_8$
formula wt	2067.86
T (K)	173 (2)
Wavelength (Å)	1.54178
crystal size (mm)	0.200 x 0.180 x 0.160
crystal syst	Triclinic
space group	P-1
a (Å)	10.2430 (6)
b (Å)	10.7700 (7)
c (Å)	25.3140 (15)
α (deg)	79.884 (3)
β (deg)	84.174 (3)
γ (deg)	62.433 (3)
V (Å^3)	2436.4 (3)
Z / Dcalcd (mg/m^3)	1/1.409
μ (mm^{-1})	5.088
F (000)	1068
Data / restraints / parameters	8892 / 2 / 574
final *R* indices [$I > 2\theta(I)$]	R1 = 0.0901
	wR2 = 0.2537
R indices (all data)	R1 = 0.1083
	wR2 = 0.2728

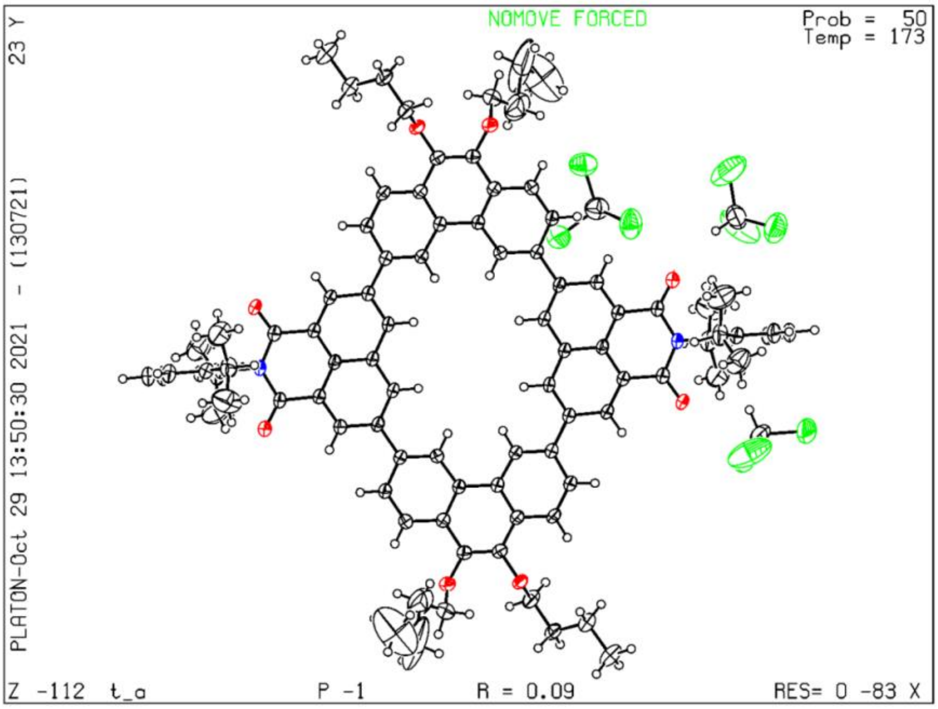

Figure S1. ORTEP diagram for the crystal structure of **NP2a** with an ellipsoid contour probability level of 50%.

3. DFT calculations.

All results presented in this report have been obtained for a molecular model where the n-butyl substituents are replaced by methyl groups. Molecular geometries have been obtained through density functional theory (DFT) calculations performed with the Gaussian09 program (Gaussian 16, Revision A.03) at the UCAM-B3LYP/6-31G(d,p) level (Becke, 1993), (Lee et al., 1988).

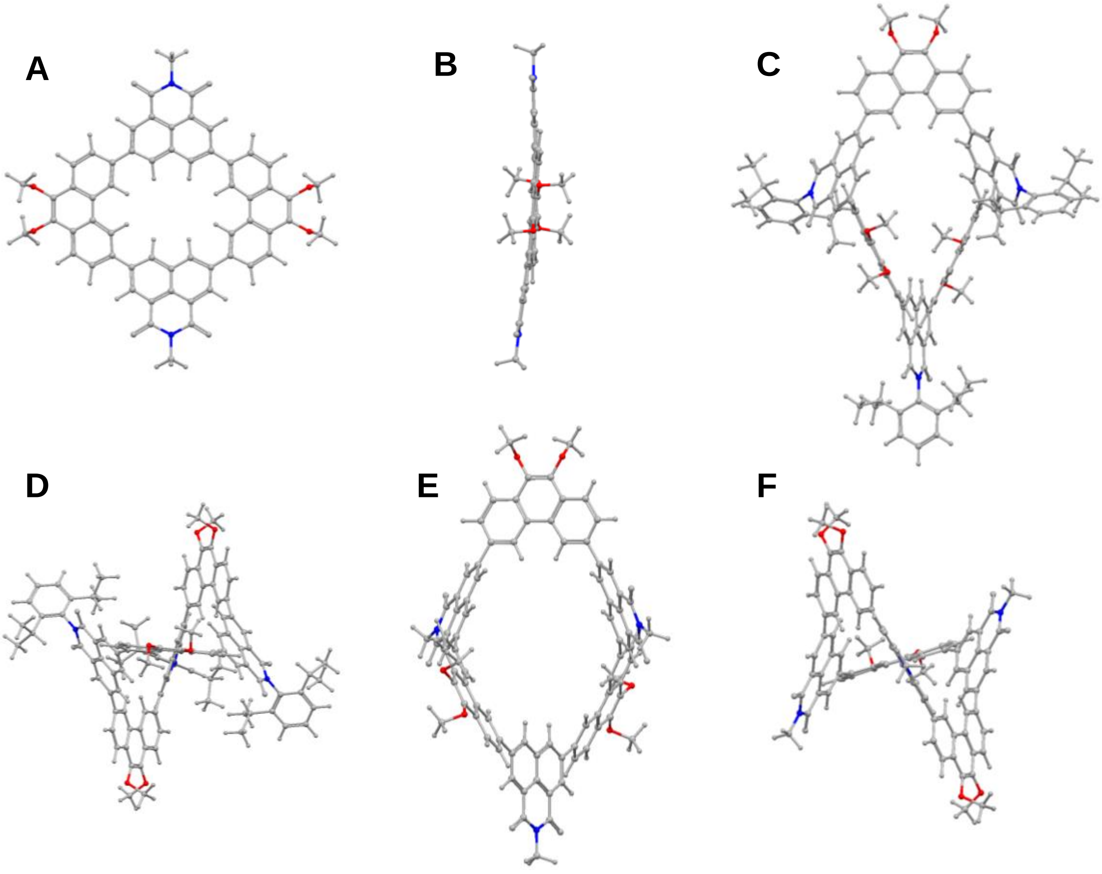

Figure S2. Calculated optimized structures: (**A**) top-view, (**B**) side-view for **NP2b**; (**C**) top-view, (**D**) side-view for **NP3a**; (**E**) top-view, (**F**) side-view for **NP3b**.

4. Additional Figures

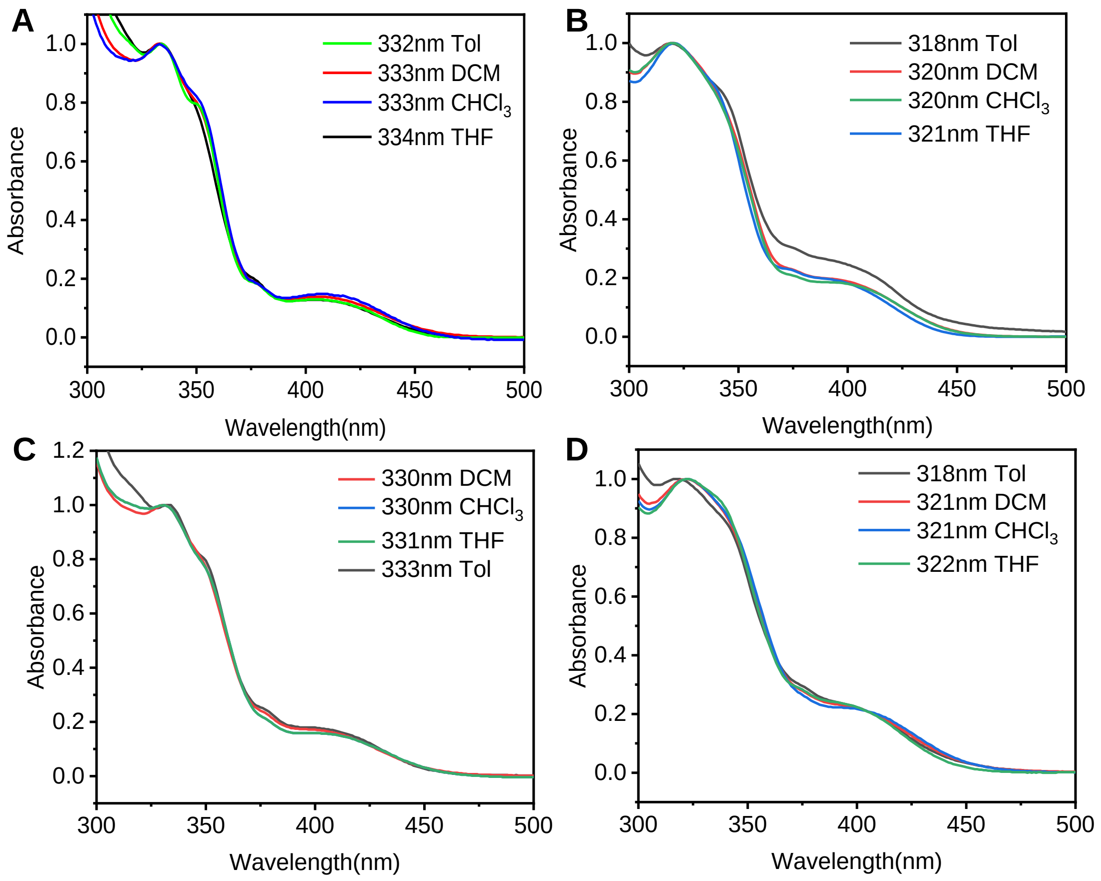

Figure S3. UV−vis absorption spectra of macrocycles in different solvents: (**A**) **NP2a**, (**B**) **NP3a**, (**C**) **NP2b** and (**D**) **NP3b.**

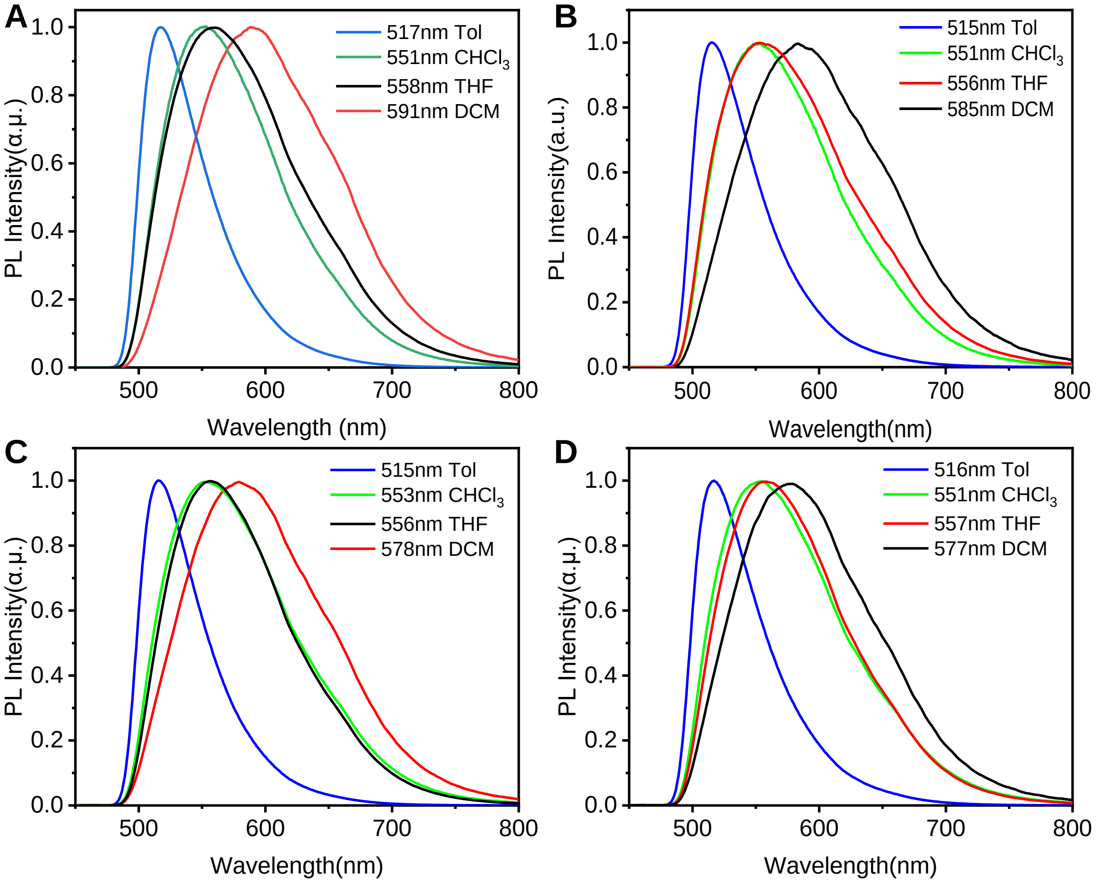

Figure S4. PL spectra of macrocycles in different solvents: (**A**) **NP2a**, (**B**) **NP3a**, (**C**) **NP2b** and (**D**) **NP3b.**

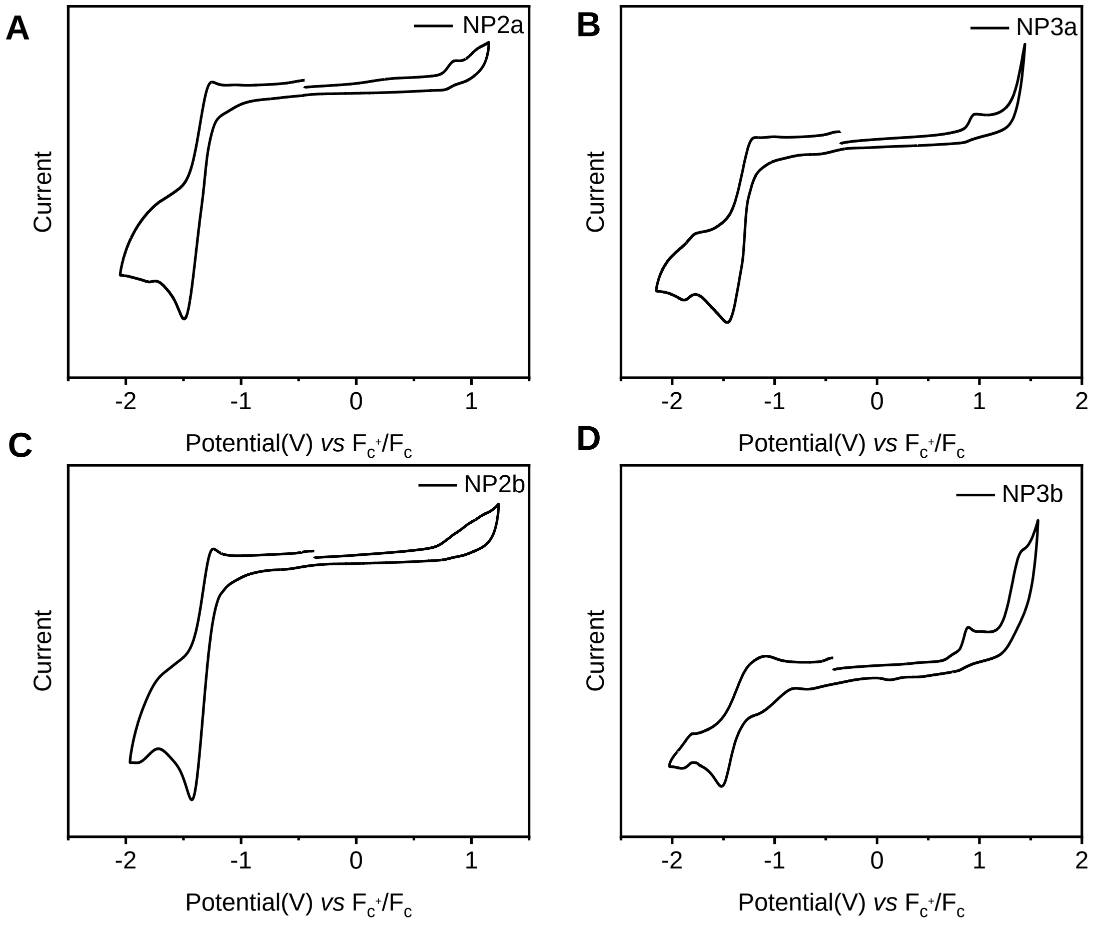

Figure S5. Cyclic voltammogram of (**A**) **NP2a**, (**B**) **NP3a**, (**C**) **NP2b** and (**D**) **NP3b** measured in anhydrous DCM with n-Bu_4NPF_6 as the supporting electrolyte.

Estimation of the association constant K_{assoc} in self-assembly:

Prepare a series of different concentrations of **NP2a**, **NP3a**, **NP2b** and **NP3b** in $CDCl_3$ solution respectively. The change of characteristic peaks in ^1H NMR spectra was monitored. The association constant K_{assoc} in self-assembly was derived by using the non-linear curve fitting based on the equation:

$$\delta = \delta_2 - \frac{2(\delta_2 - \delta_0)}{1 + \sqrt{1 + 8cK_{assoc}}}$$

Where δ is the weighted average chemical shift at different concentrations of macrocycle (c), δ_0 is the chemical shift of the monomer and δ_2 is the chemical shift of the dimer.

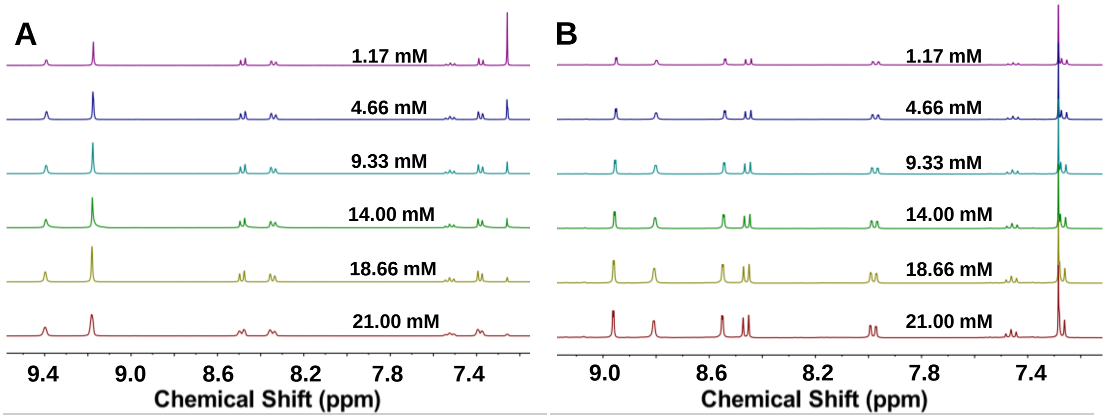

Figure S6. ^1H NMR (in $CDCl_3$) spectroscopy at different concentrations for (**A**) **NP2a**, (**B**) **NP3a**.

Estimation of the binding constant K_a with C_{70} in toluene:

To determine the stoichiometry and association constant for the complexation between macrocycles and C_{70}, fluorescence titration experiments were done in which the solution of C_{70} in toluene was added to a solution of **NP2a**, **NP3a**, **NP2b** and **NP3b** at 25 °C, respectively. The change in the maximum fluorescence emission peak at 516 nm was monitored. By a mole ratio plot, 1:1 stoichiometry was obtained for the complexation between NP2a/NP3a/NP2b/NP3b and C_{70}. And the association constant K_a for the 1:1 complexes was derived by using the non-linear curve fitting based on the equation: $\Delta I = \Delta I_\infty((1 + K_a[G] + K_a[H]_0) - ((1 + K_a[G] + K_a[H]_0)^2 - 4 K_a^2[H]_0[G])^{0.5}) /(2K_a[H]_0)$, Where $\Delta I = I - I_0$, $\Delta I_\infty = I_\infty - I_0$, [G] is [$C_{70}$], $[H]_0$ = [**NP2a**/**NP3a**/**NP2b**/**NP3b**].

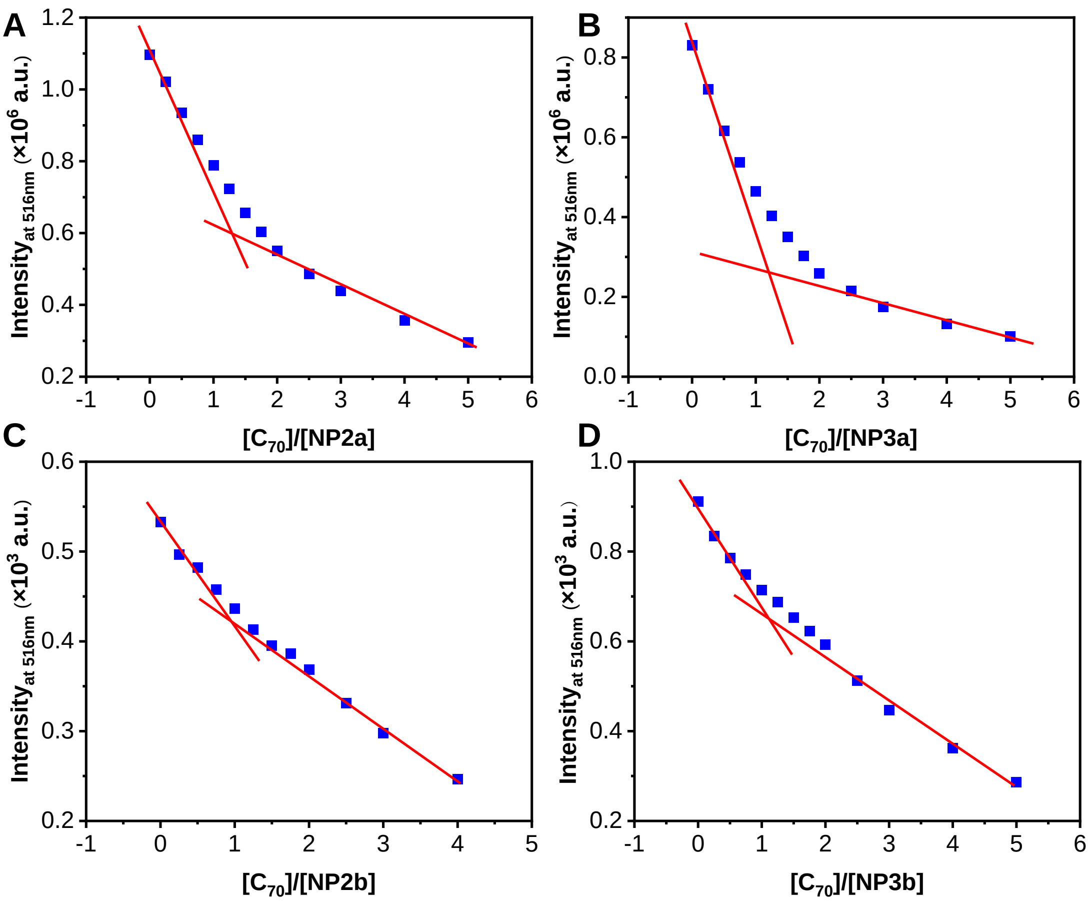

Figure S7. Mole ratio plot for C_{70} and (**A**) **NP2a**, (**B**) **NP3a**, (**C**) **NP2b**, (D) **NP3b**. All the plots show a 1:1 complexation stoichiometry.

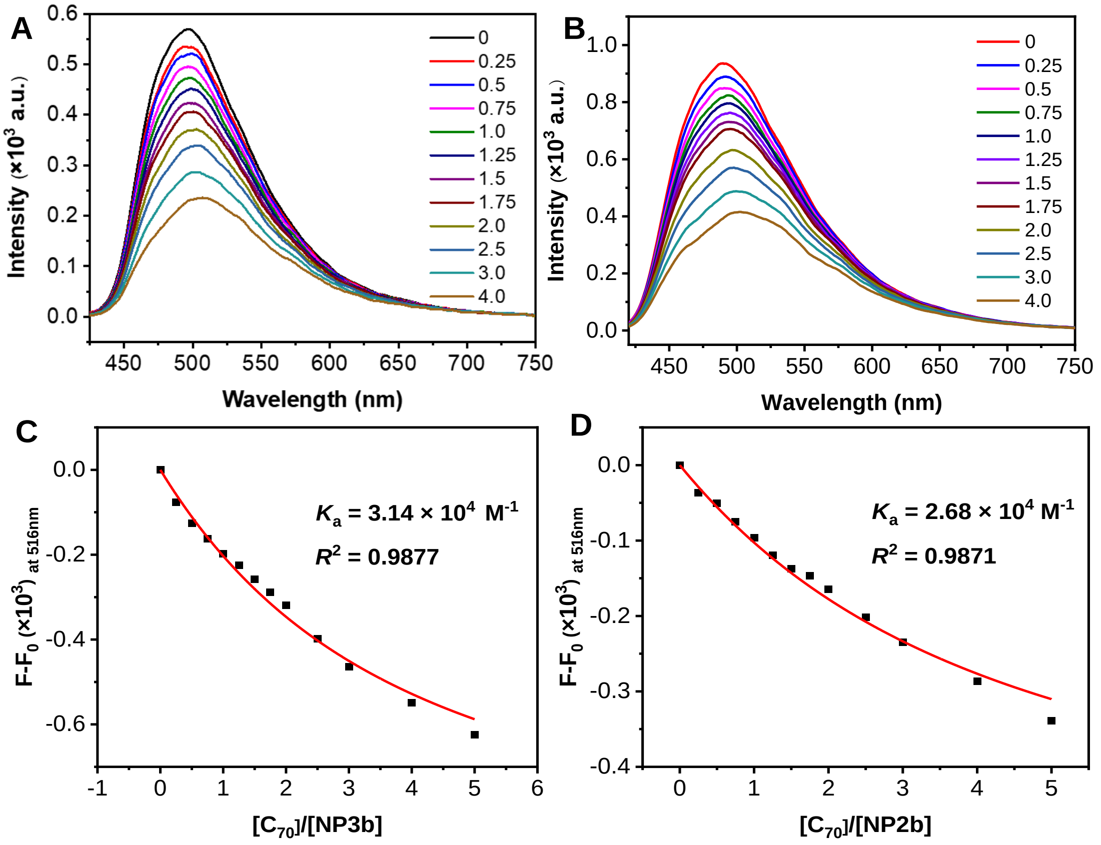

Figure S8. Fluorescence spectral change of (**A**) **NP2b** and (**B**) **NP3b** during titration with C_{70}. Fitting curves on the relative fluorescent intensity of (**C**) **NP2b**, (**D**) **NP3b** for obtaining K_a, R^2 is the coefficient of determination.

Estimation of the binding constant K_a with electron-rich guest pyrene:

To determine the stoichiometry and association constant between macrocycles and pyrene, ^{1}H NMR titration was done with $CDCl_3$ solutions which had a constant concentration of macrocycles (2.5 mM) and varying concentrations of pyrene. By mole ratio plots, a 1:1 stoichiometry was obtained, suggesting that the macrocycles **NP2a/NP2b** was shown to form a 1:1 complex with pyrene. Titration curve-fitting and association constant values were calculated by employing the BindFit program developed by Prof. Pall Thordarson of UNSW (Thordarson, 2011) and 1:1 binding stoichiometry was chosen in the BindFit program. This program employs a nonlinear least-squares regression analysis and is available free of cost online through the following link: http://supramolecular.org.

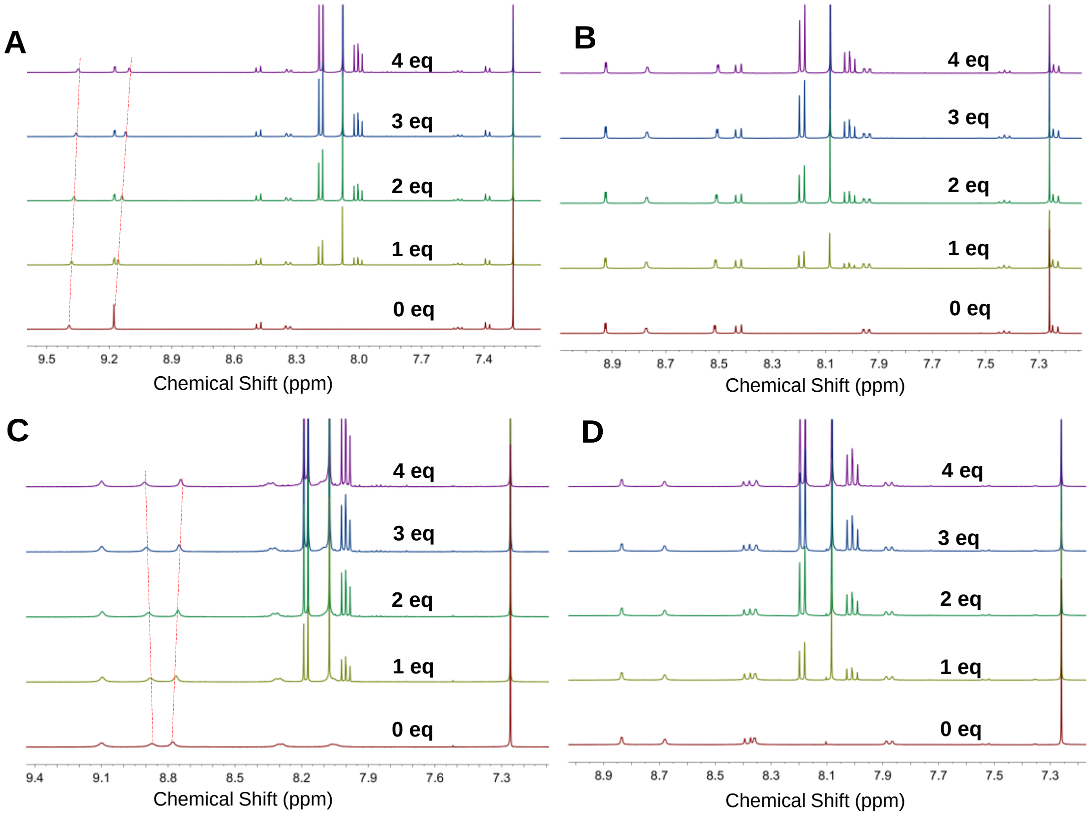

Figure S9. ^{1}H NMR spectra of different concentrations of pyrene added to the $CDCl_3$ solution of macrocycle. (**A**) **NP2a**, (**B**) **NP3a**, (**C**) **NP2b** and (D) **NP3b**.

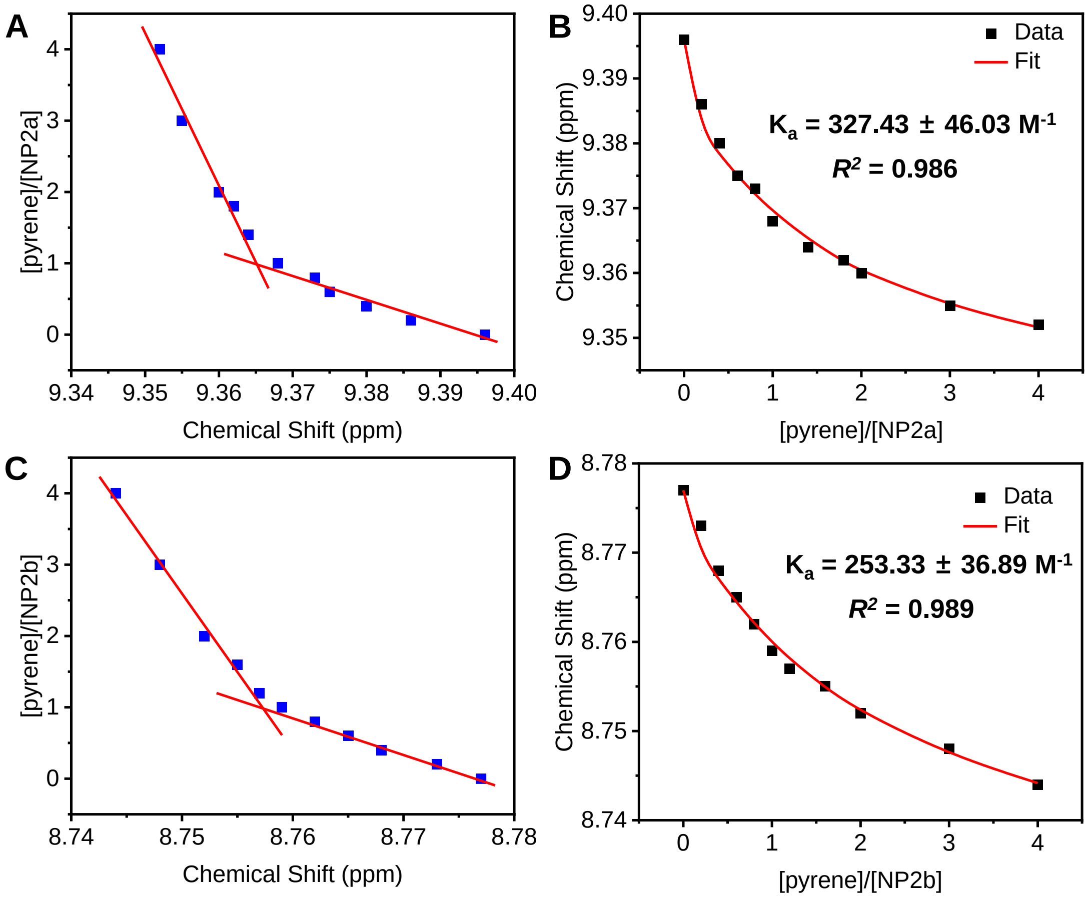

Figure S10. Mole ratio plot of the complexation of pyrene and (**A**) **NP2a**, (**C**) **NP2b in $CDCl_3$ at 298 K.** Fitting binding isotherm using a 1:1 association model for the titration of (**B**) **NP2a**, (**D**) **NP2b** with pyrene.

5. ^{1}H, ^{13}C NMR and high resolution mass spectrometry

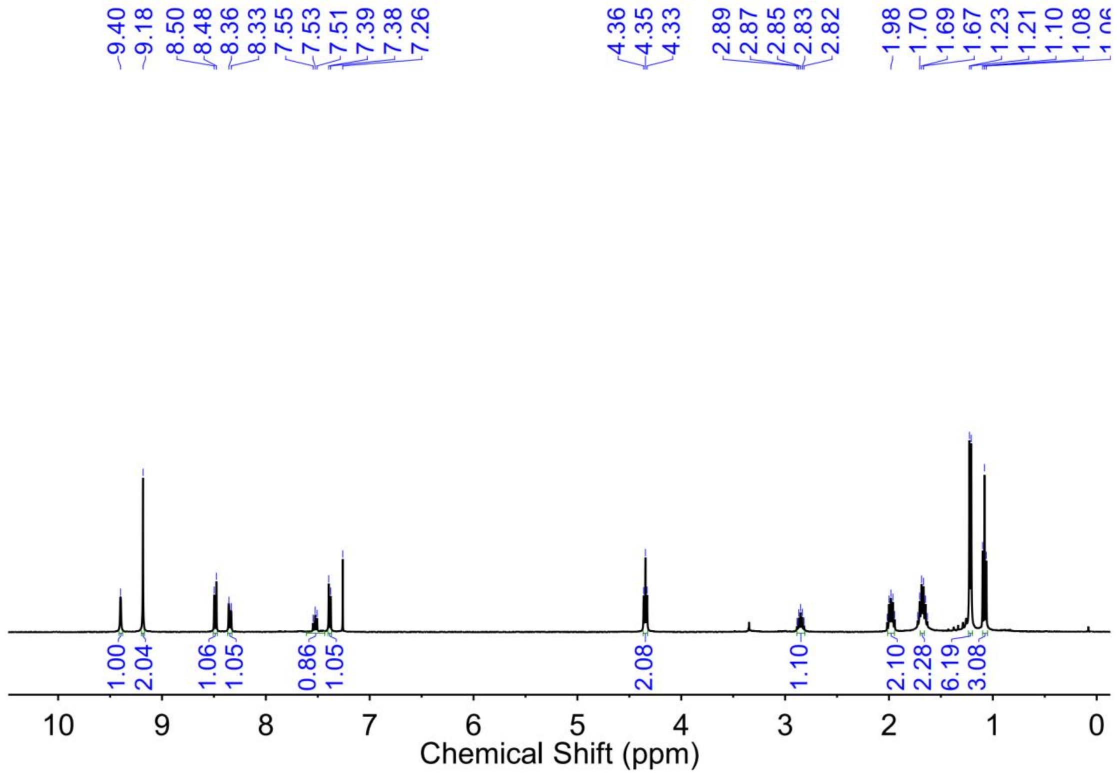

Figure S11. ^{1}H NMR spectrum (400 MHz) of **NP2a** in $CDCl_3$ at 298 K.

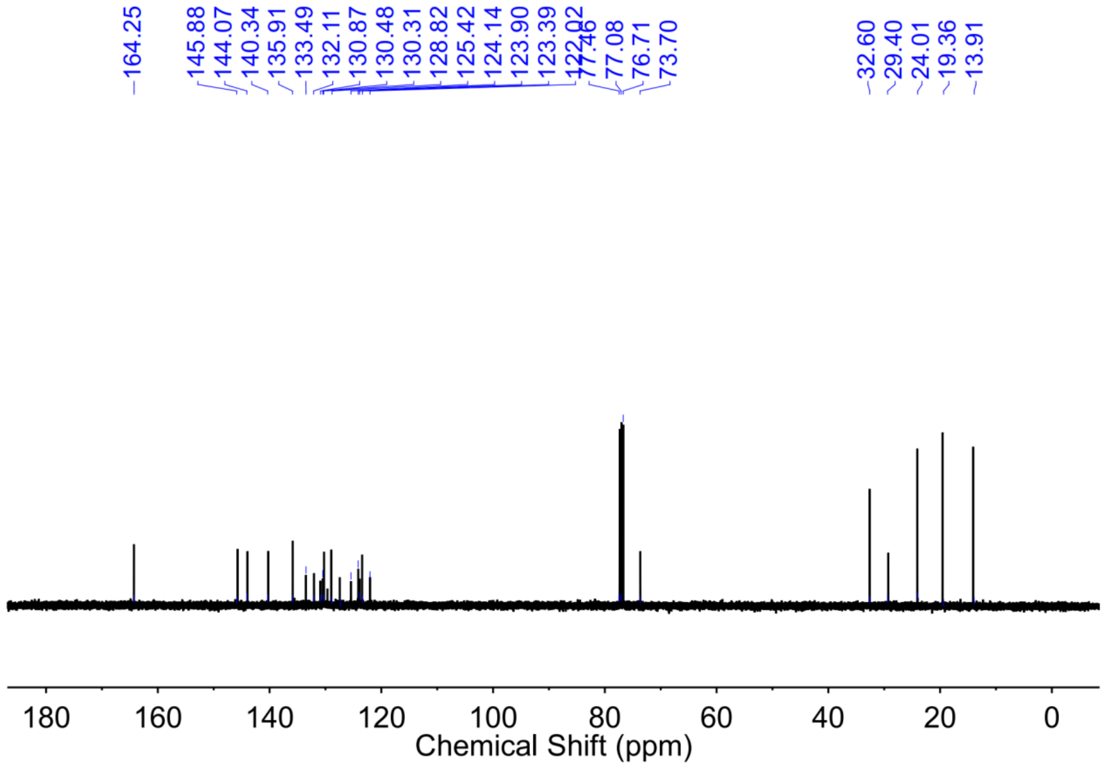

Figure S12. ^{13}C NMR spectrum (100 MHz) of **NP2a** in $CDCl_3$ at 298 K.

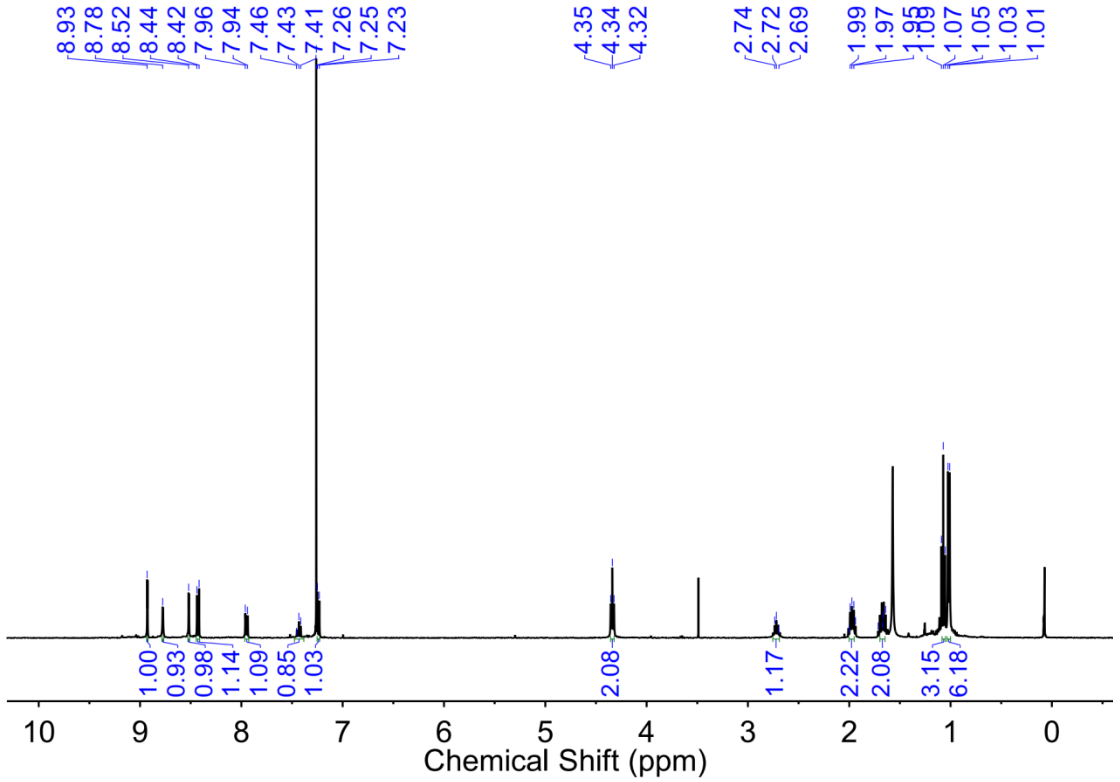

Figure S13. ^{1}H NMR spectrum (400 MHz) of **NP3a** in $CDCl_3$ at 298 K.

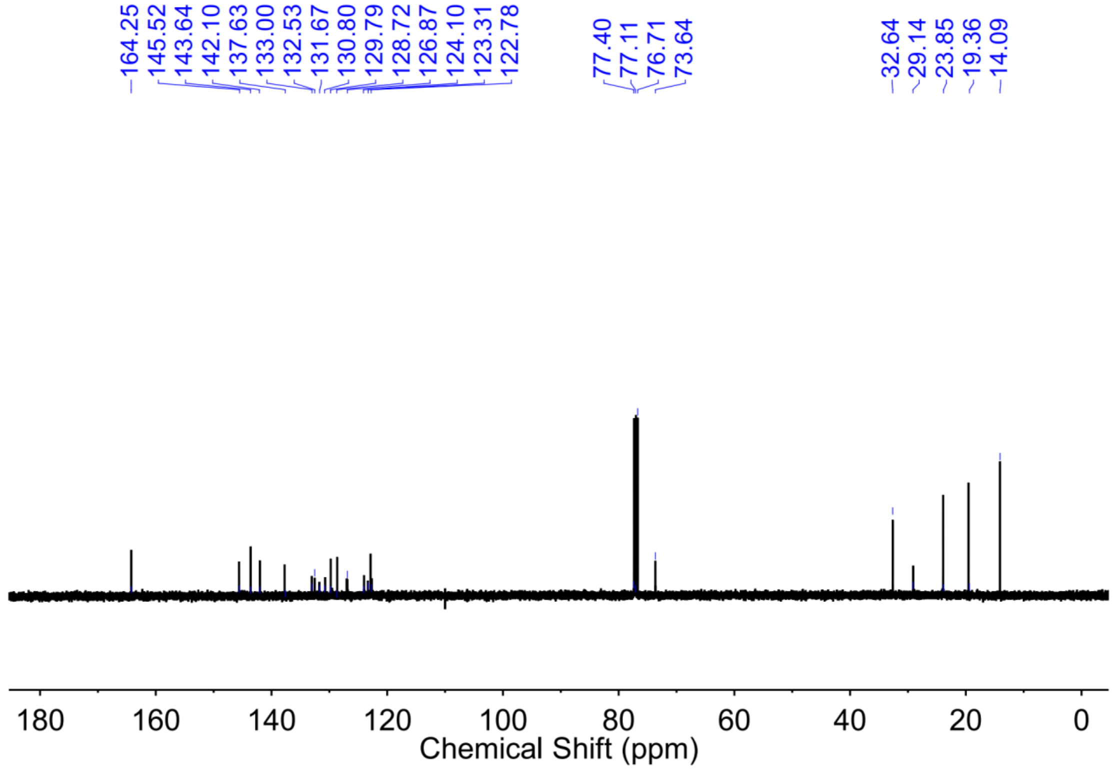

Figure S14. ^{13}C NMR spectrum (100 MHz) of **NP3a** in $CDCl_3$ at 298 K.

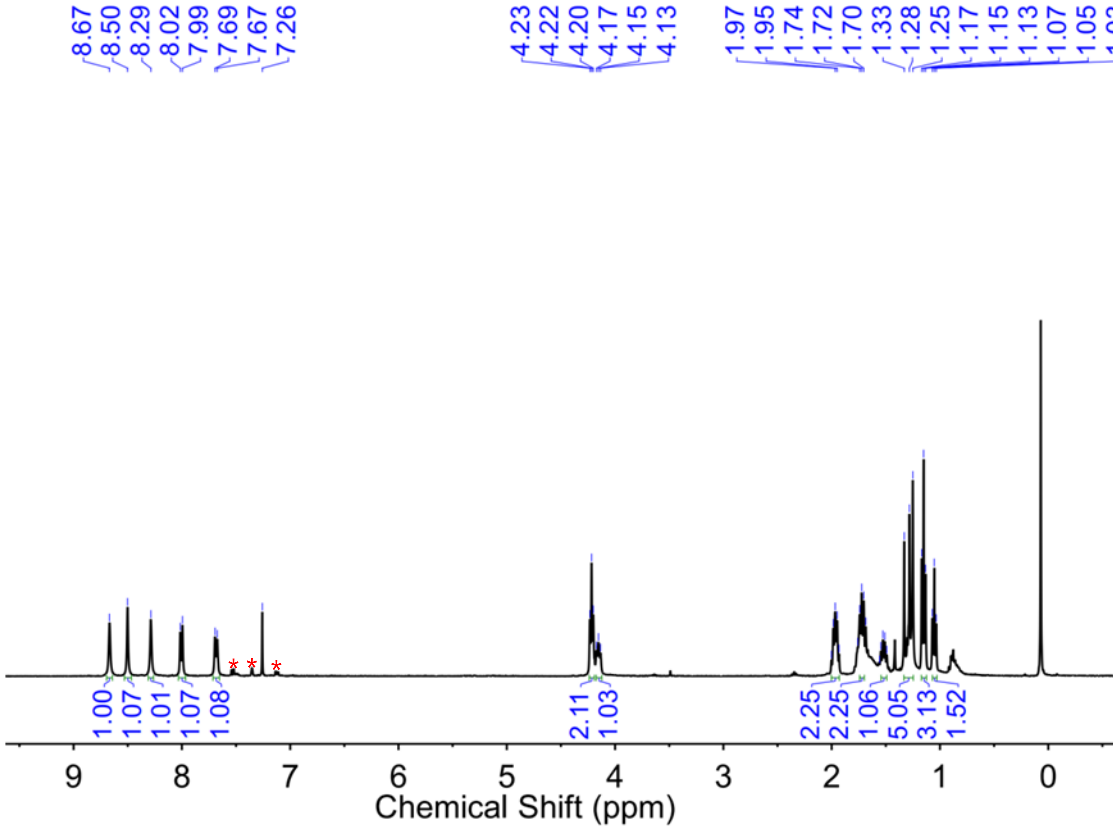

Figure S15. ^{1}H NMR spectrum (400 MHz) of **NP2b** in $CDCl_3$ at 298 K.

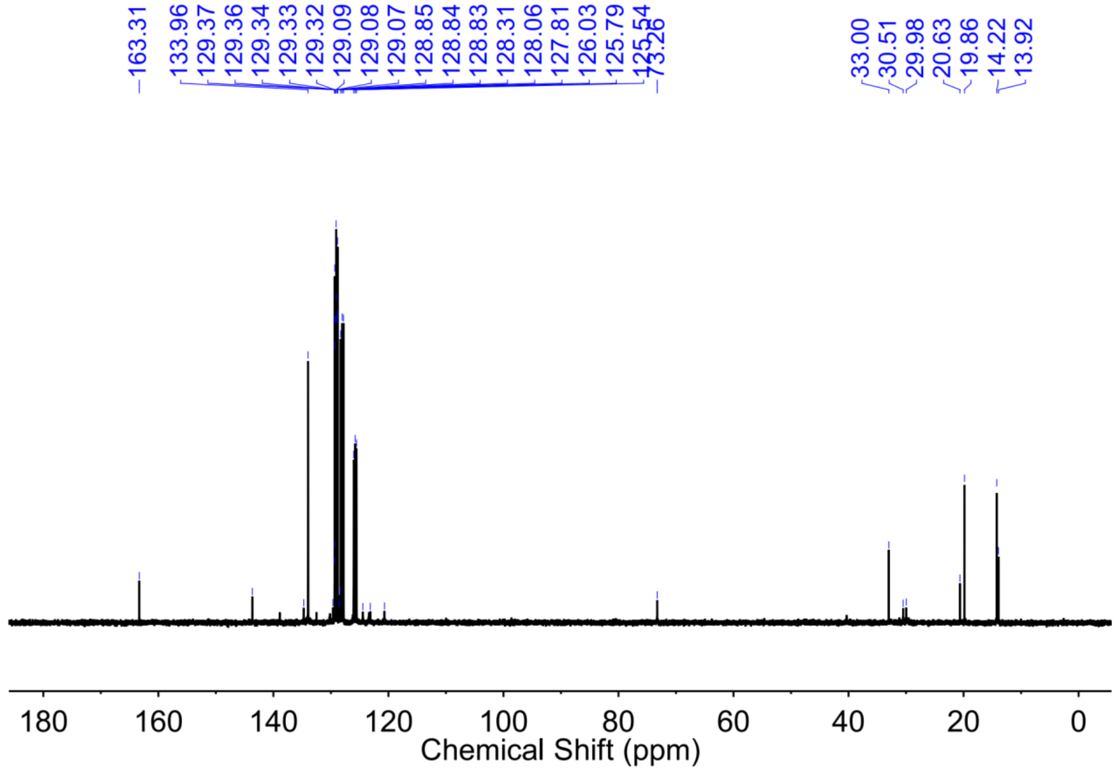

Figure S16. ^{13}C NMR spectrum (100 MHz) of **NP2b** in $CDCl_3$ at 298 K.

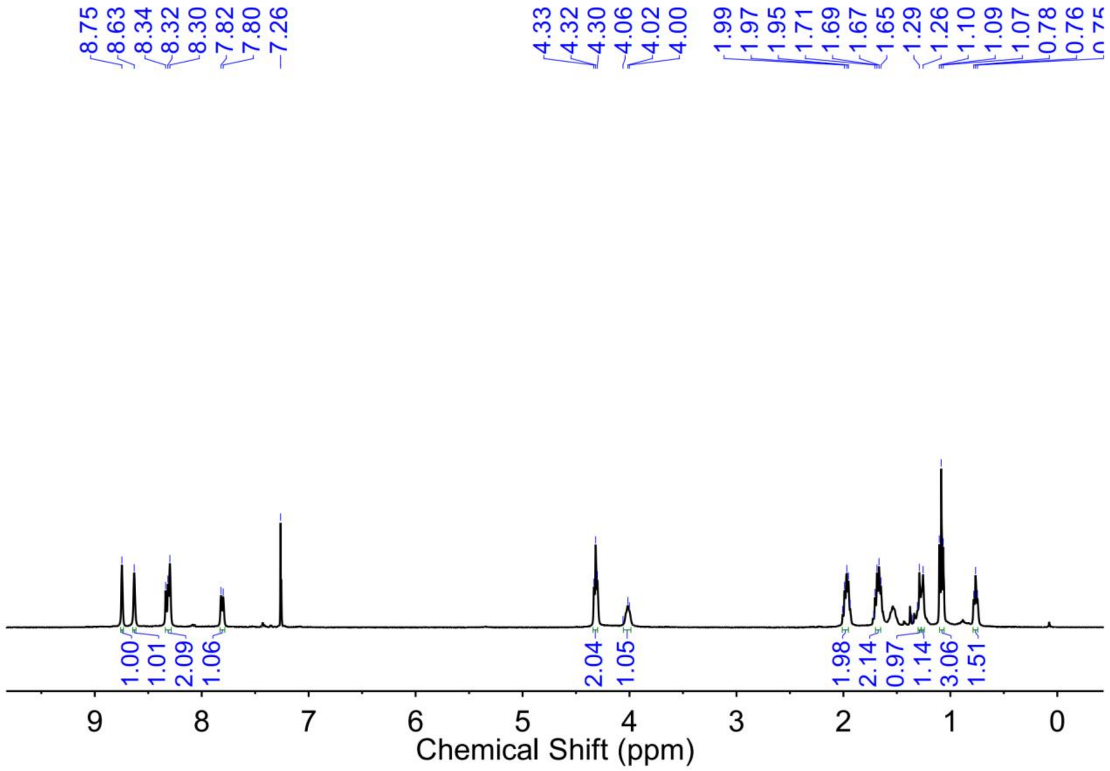

Figure S17. ^{1}H NMR spectrum (400 MHz) of **NP3b** in $CDCl_3$ at 298 K.

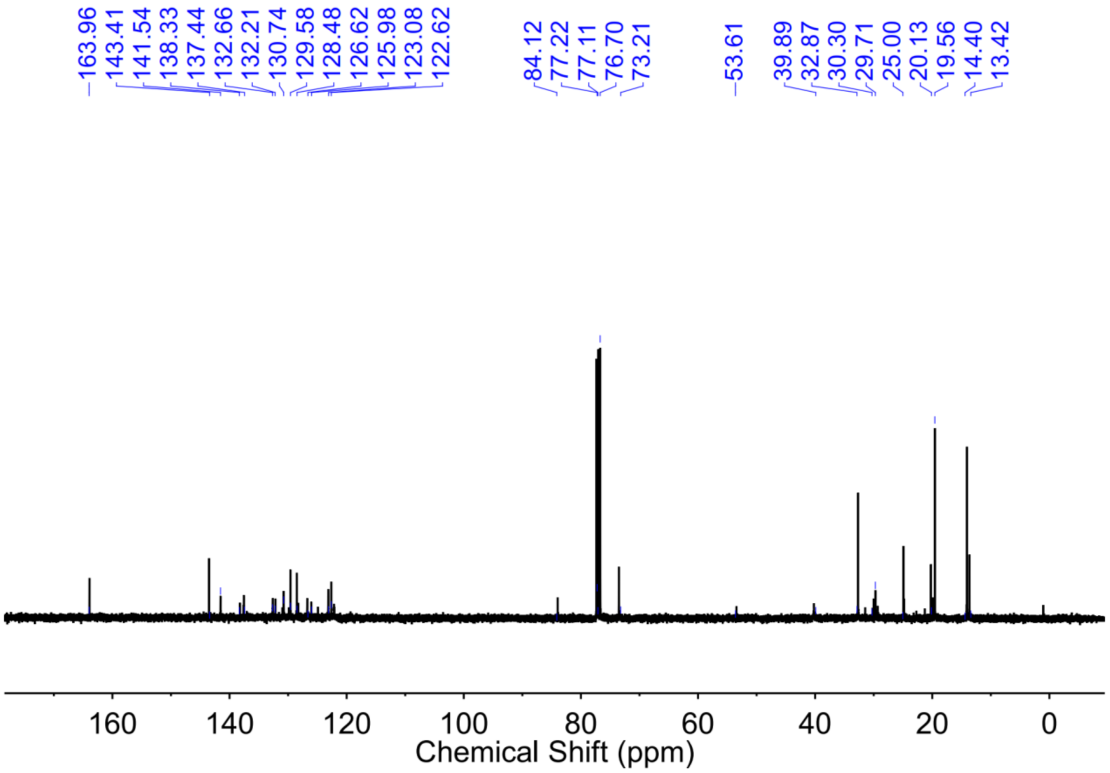

Figure S18. ^{13}C NMR spectrum (100 MHz) of **NP3b** in $CDCl_3$ at 298 K.

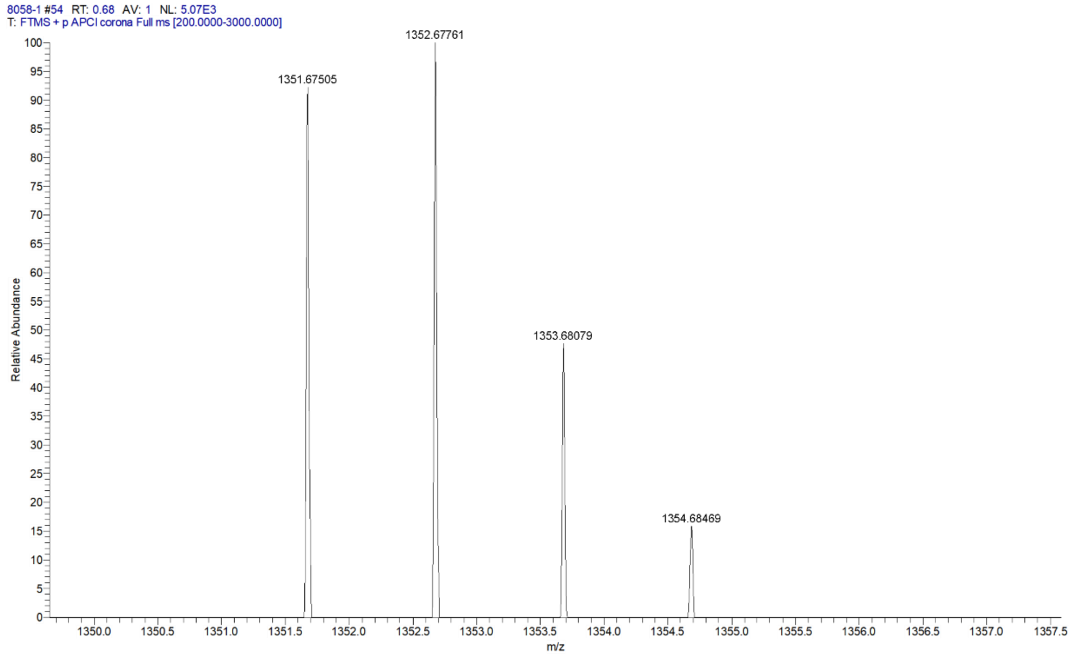

Figure S19. HR mass spectrum (APCI) of **NP2a**.

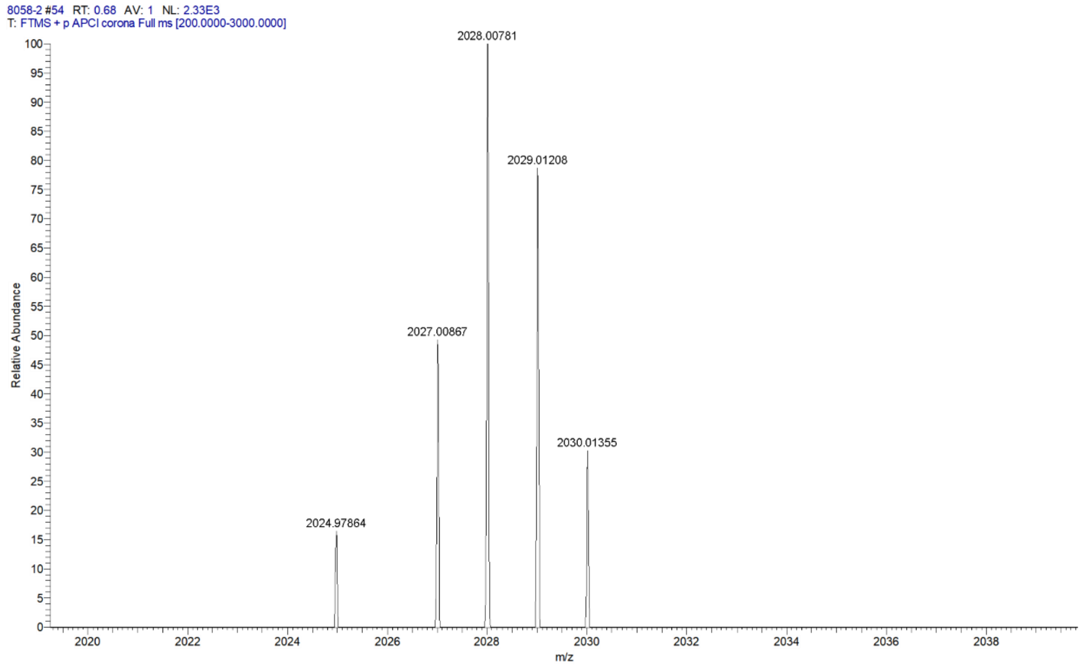

Figure S20. HR mass spectrum (APCI) of **NP3a**.

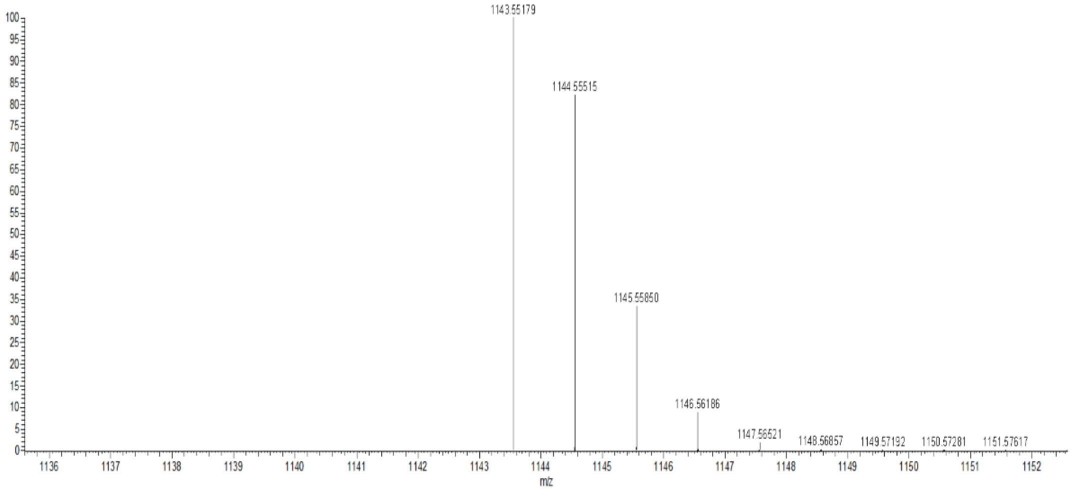

Figure S21. HR mass spectrum (APCI) of **NP2b**.

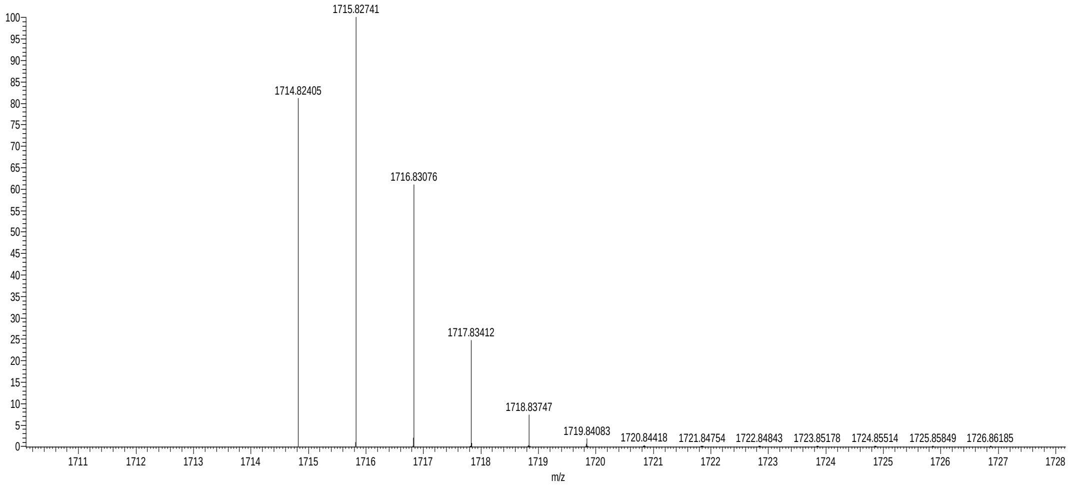

Figure S22. HR mass spectrum (APCI) of **NP3b**.

6. References

Becke, A.D. (1993). Density-functional thermochemistry. III. The role of exact exchange. *J. Chem. Phys.* 98(7), 5648-5652. doi: 10.1063/1.464913.

Gaussian 16, Revision A.03. (2016). M. J. Frisch, G. W. Trucks, H. B. Schlegel, G. E. Scuseria, M. A. Robb, J. R. Cheeseman, G. Scalmani, V. Barone, G. A. Petersson, H. Nakatsuji, X. Li, M. Caricato, A. V. Marenich, J. Bloino, B. G. Janesko, R. Gomperts, B. Mennucci, H. P. Hratchian, J. V. Ortiz, A. F. Izmaylov, J. L. Sonnenberg, D. Williams-Young, F. Ding, F. Lipparini, F. Egidi, J. Goings, B. Peng, A. Petrone, T. Henderson, D. Ranasinghe, V. G. Zakrzewski, J. Gao, N. Rega, G. Zheng, W. Liang, M. Hada, M. Ehara, K. Toyota, R. Fukuda, J. Hasegawa, M. Ishida, T. Nakajima, Y. Honda, O. Kitao, H. Nakai, T. Vreven, K. Throssell, J. A. Montgomery, Jr., J. E. Peralta, F. Ogliaro, M. J. Bearpark, J. J. Heyd, E. N. Brothers, K. N. Kudin, V. N. Staroverov, T. A. Keith, R. Kobayashi, J. Normand, K. Raghavachari, A. P. Rendell, J. C. Burant, S. S. Iyengar, J. Tomasi, M. Cossi, J. M. Millam, M. Klene, C. Adamo, R. Cammi, J. W. Ochterski, R. L. Martin, K. Morokuma, O. Farkas, J. B. Foresman, and D. J. Fox, Gaussian, Inc., Wallingford CT.

Gregolińska, H., Majewski, M., Chmielewski, P.J., Gregoliński, J., Chien, A., Zhou, J., et al. (2018). Fully Conjugated [4]Chrysaorene. Redox-Coupled Anion Binding in a Tetraradicaloid Macrocycle. *J. Am. Chem. Soc.* 140(43), 14474-14480. doi: 10.1021/jacs.8b09385.

Kiyotaki, K., Kayukawa, T., Imayoshi, A., and Tsubaki, K. (2020). Total Syntheses of FR-901235, Auxarthrones A–D, and Lamellicolic Anhydride. *Org. Lett.* 22(23), 9220-9224. doi: 10.1021/acs.orglett.0c03401.

Lee, C., Yang, W., and Parr, R.G. (1988). Development of the Colle-Salvetti correlation-energy formula into a functional of the electron density. *Phys. Rev. B.* 37(2), 785-789. doi: 10.1103/PhysRevB.37.785.

Lu, X., Gopalakrishna, T.Y., Phan, H., Herng, T.S., Jiang, Q., Liu, C., et al. (2018). Global Aromaticity in Macrocyclic Cyclopenta-Fused Tetraphenanthrenylene Tetraradicaloid and Its Charged Species. *Angew. Chem. Int. Ed.* 57(40), 13052-13056. doi: 10.1002/anie.201807185.

Phulwale, B.V., Mishra, S.K., Nečas, M., and Mazal, C. (2016). Phenanthrylene-butadiynylene and Phenanthrylene-thienylene Macrocycles: Synthesis, Structure, and Properties. *J. Org. Chem.* 81(15), 6244-6252. doi: 10.1021/acs.joc.6b00814.

Thordarson, P. (2011). Determining Association Constants from Titration Experiments in Supramolecular Chemistry. *Chem. Soc. Rev.* 2011, 40, 1305-1323. doi: 10.1039/C0CS00062K.

Xue, J.Y., Nakanishi, W., Tanimoto, D., Hara, D., Nakamura, Y., and Isobe, H. (2013). Convergent synthesis of hexameric naphthylene macrocycles with dicarboxylic imide appendages. *Tetrahedron Letters.* 54(36), 4963-4965. doi: 10.1016/j.tetlet.2013.07.025.
